# TYRP1 mRNA expression in melanoma metastases correlates with clinical outcome

**DOI:** 10.1038/bjc.2011.451

**Published:** 2011-11-01

**Authors:** F Journe, H Id Boufker, L Van Kempen, M-D Galibert, M Wiedig, F Salès, A Theunis, D Nonclercq, A Frau, G Laurent, A Awada, G Ghanem

**Affiliations:** 1 Laboratoire d’Oncologie et de Chirurgie Expérimentale, Institut Jules Bordet, Université Libre de Bruxelles, 1 rue Heger-Bordet, B1000, Brussels, Belgium; 2 Department of Pathology, Radboud University Nijmegen Medical Centre, Nijmegen, The Netherlands; 3 CNRS UMR 6061, Institut de Génétique et Développement de Rennes, Rennes, France; 4 Département de Pathologie, Institut Jules Bordet, Université Libre de Bruxelles, Brussels, Belgium; 5 Service d’Histologie, Université de Mons, Mons, Belgium; 6 Clinique d’Oncologie Médicale, Institut Jules Bordet, Université Libre de Bruxelles, Brussels, Belgium

**Keywords:** melanoma, prognosis, marker, Breslow, survival

## Abstract

**Background:**

Clinical outcome of patients with high-risk melanoma cannot be reliably predicted on the basis of classical histopathological examination. Our study aimed to determine in melanoma metastases a gene expression profile associated with patient survival, and to identify and validate marker(s) of poor clinical outcome.

**Methods:**

Skin and lymph node metastases from melanoma patients (training population) were used to identify candidate prognostic marker(s) based on DNA microarray analysis. Additional skin metastases (validation population) were used to assess the prognostic value of the first ranked gene by real-time PCR.

**Results:**

We performed microarray analysis in the training population and generated a list of 278 probe sets associated with a shorter survival. We used the first ranked gene, *tyrosinase-related protein 1* (*TYRP1*), further measured its expression in the validation population by real-time PCR and found it to be significantly correlated with distant metastasis-free survival (DMFS), overall survival (OS) and Breslow thickness. We also found that it was fairly well conserved in the course of the disease regardless of the delay to metastasis occurrence. Finally, although Tyrp1 protein (immunohistochemistry (IHC)) was only detected in about half of the samples, we showed that its expression also correlated with Breslow thickness.

**Conclusion:**

Our data indicate that TYRP1 mRNA expression level, at least in skin metastases, is a prognostic marker for melanoma, and is particularly useful when prognostic pathology parameters at the primary lesion are lacking. Its conserved expression further supports its use as a target for therapy.

The prognosis of melanoma is based on the histopathological criteria described in the American Joint Committee on Cancer (AJCC) melanoma staging system. These include the Breslow index, mitotic rate, ulceration status and extent of lymph node involvement (Balch *et al*, 2009). Despite this staging system, the clinical behaviour of melanoma is often unpredictable (Nagore *et al*, 2005), because melanoma is a group of diseases with various biological subtypes (Lomas *et al*, 2008). In addition, patients with melanoma metastatic to the skin show variable survival: some may survive for a long time, whereas some die of disseminated disease within 1 year of removal of skin metastases (Hofmann-Wellenhof *et al*, 1996).

Studies based on melanoma gene expression profiling have been performed in order to improve the prognosis of the disease and to predict its response to treatment (Hoek, 2007). First, a comparison of gene expression profiles of normal skin, nevi and primary and metastatic melanomas has identified 2602 signature genes that could be used to distinguish two metastatic patterns, which are already emergent in large primaries (Haqq *et al*, 2005). Second, cDNA expression microarray in primary melanoma has revealed a signature of 254 genes characterising patients at risk of developing distant metastases (Winnepenninckx *et al*, 2006). Third, high-throughput gene microarray in metastatic melanoma has determined a set of 80 probes (70 genes) associated with survival (Mandruzzato *et al*, 2006). Finally, molecular profiling of lymph node metastases of stage III melanoma patients has disclosed 21 genes whose expression levels correlated with clinical outcome (John *et al*, 2008). Thus, several new marker genes have shown promise, and large-scale studies are now warranted to clinically validate them for the development of new prognostic tools, diagnostic approaches and biological-targeted therapies (Larson *et al*, 2009).

Such gene discovery platforms may help to identify new molecular markers in melanoma metastases, enabling one to refine the prognosis at the time of tumour progression, especially in thin melanomas. They may also help to establish a prognosis in patients with unknown melanoma primaries (2–6% of all melanoma cases) (Schlagenhauff *et al*, 1997). The identification of such markers in high-risk melanoma patients would be important for the design and the interpretation of clinical trials, and could be of great benefit as one might also foresee the development of useful and effective adjuvant therapies.

This work describes a gene profiling study in skin and lymph nodes, which are the most frequent melanoma metastases, that shows an inverse correlation between tyrosinase-related protein 1 (TYRP1) expression level and patient overall survival. A validation study using quantitative PCR only in skin metastases further supports TYRP1 as a new marker of poor clinical outcome.

## Materials and methods

### Patients and tissue collection

Skin and lymph node metastases were collected from patients with stage III melanoma undergoing surgery at Institut Jules Bordet. Samples (mean size 10 mm, no necrosis) were collected randomly with no inclusion or exclusion criteria. Half of each biopsy was fixed in formalin, embedded in paraffin, sectioned according to routine clinical procedures and was used for immunohistochemistry. The other half was snap-frozen in liquid nitrogen and stored at −80 °C, and was dedicated to microarray analysis and real-time PCR. Microdissection has been carried out by one surgeon (FS) on each sample before any snap freezing. This study was approved by the ethic committee of Institut Jules Bordet and performed in accordance with the REMARK guidelines (Alonzo, 2005; McShane *et al*, 2005). The clinical characteristics of the patients are outlined in Table 1.

### RNA extraction

Frozen samples were homogenised using the FastPrep-24 homogeniser system with lysing matrix D (MP Biomedicals, Illkirch Cedex, France) in RLT buffer supplemented with *β*-mercaptoethanol (RNeasy Mini Kit, Qiagen, Venlo, The Netherlands) at 4 °C. Centrifugation with RNeasy spin column separated melanin from the total RNA. After washing steps, RNA was collected in RNase-free water and RNA concentrations were evaluated using a NanoDropTM 1000 spectrophotometer (Thermo Scientific, Wilmington, DE, USA). RNA quality of each sample was assessed based on the RNA profile generated by the bioanalyzer (Agilent Technologies, Santa Clara, CA, USA).

### Microarray analysis

Gene expression profiling was performed with the training population using the Affymetrix technology (Affymetrix, Inc., Santa Clara, CA, USA). RNA was hybridised on Human Genome U133 Plus 2.0 Array. The scanning of the chips was done according to standard Affymetrix protocols. Image analysis and probe quantification were performed with the Affymetrix software that produced raw probe intensity data in the Affymetrix CEL files. Before statistical analysis, data were loaded and normalised (RMA program) using the software package BRB Array Tools (http://linus.nci.nih.gov/BRB-ArrayTools.html). A class comparison was performed between groups of arrays based on patient survival (group 1 (*N*=10): overall survival (OS) <30 months; group 2 (*N*=22): OS ⩾30 months) sorting probe sets passing filtering criteria (significant at 0.05 level of the univariate two-sample *t*-test and fold change strictly >2.5). To assess a possible bias due to a difference in tumour burden, the expression of the specific melanocyte marker S100B was compared between both groups.

### Real-time PCR

The TYRP1 mRNA expression was quantified by real-time PCR. cDNA was synthesised using a standard reverse transcription method (qScript cDNA SuperMix, Quanta Biosciences, Gaithersburg, MD, USA). Real-time PCR reactions were performed using the SYBR Green PCR Master Mix (Applied Biosystems, Foster City, CA, USA) and sequence-specific primer sets for TYRP1 (forward=5′-CCGAAACACAGTGGAAGGTT-3′, reverse=5′-TCTGTGAAGGTGTGCAGGA-3′), for S100B (forward=5′-ATTCTGGAAGGGAGGGAGAC-3′, reverse=5′-CGTGGCAGGCAGTAGTAACC-3′) and for *β*-actin (forward=5′-CTGGCACCCAGCACAATG-3′, reverse=5′-CCGATCCACACGGAGTACTTG-3′) (Sigma-Genosys, Pampisford Cambs, UK). The amplification was performed on an ABI PRISM 7900HT Sequence Detection System (Applied Biosystems) using 40 cycles of a two-step PCR (15 s at 95 °C and 60 s at 60 °C) after an initial activation step (95 °C for 10 min). Melting curves from 60 °C to 99 °C were assessed to evaluate PCR specificity. Serial dilutions of purified amplicons were utilised to generate standard melting curves. The mRNA expressions of TYRP1 and S100B were normalised to *β*-actin (loading control). The S100B is one of the most reliable biomarkers for melanoma and it is commonly used as a marker for tumour load.

### Immunohistochemistry

For Tyrp1 immunostaining, dewaxed tissue sections were rehydrated in distilled water, incubated for 1 h in distilled water at 85 °C, and exposed for 5 min to 0.5% H_2_O_2_. Thereafter, the sections were rinsed in phosphate-buffered saline (PBS), incubated for 15 min in PBS containing 0.5% casein, and then exposed for 1 h to a primary antibody raised against the carboxyterminal end of Tyrp1 protein (mouse monoclonal anti-Tyrp1, dilution 1 : 50, clone G3E6; Abcam, Cambridge, UK), and for additional 30 min to a secondary antibody raised against mouse immunoglobulins and conjugated with horseradish peroxidase (HRP-conjugated goat polyclonal anti-mouse IgG, dilution 1 : 50; Abcam). Bound peroxidase activity was visualised by incubation in the presence of H_2_O_2_ and 3-amino-9-ethylcarbazole (AEC substrate) (Vector Laboratories, Burlingame, CA, USA). The sections were counterstained with hemalun, mounted using an aqueous-based medium (Vectamount AQ, Vector), and analysed as described in Figure 3.

### Statistical analysis

For statistical analyses of microarray and real-time PCR data, intensity values were log-transformed to a base-2 scale. False discovery rate (FDR) was determined using the software BRB Array Tools. Statistical correlation between two variables was assessed using Spearman's rho test. Statistical significance between two independent groups was examined using the Mann–Whitney test. Variations of TYRP1 expression during time in different patients were tested by Kruskal–Wallis test. Distant metastasis-free survival (DMFS) and OS were estimated using the Kaplan–Meier method. Univariate analyses of relapse/death were performed using Cox's proportional hazards method. Significance of the positive predictive value was determined by Fisher's exact test. The *P*-values of <0.05 were considered as statistically significant. All statistical analyses were performed using SPSS 15.0 Inc. (Chicago, IL, USA).

## Results

### Characteristics of melanoma patients

The characteristics of the training and the validation populations are in Table 1. A large majority of patients (95%) had primary melanoma with a Breslow thickness >1 mm and thus an unfavourable prognosis (however, 5 patients in the validation population had tumours with <1 mm thickness). In the training population, all patients deceased, whereas in the validation population some patients were lost for follow-up (6 out of 89) or were still alive (10 out of 89) over a period of up to 25 years.

### Identification of genes related to survival in the training population

The training population was subjected to microarray analysis as described in the Materials and Methods. A class comparison between two groups of samples from patients with different survival sorted 278 probe sets passing filtering criteria (Supplementary Table 1). The first ranked gene was *TYRP1* (fold change=33.9, *P*=0.00004), which actually codes for an enzyme involved in melanogenesis, a unique feature restricted to melanocytes. Interestingly, among the other highly ranked upregulated probe sets, five genes were also associated with the pigmentation (*SILV*, *DCT*, *OCA2*, *TYR* and *MITF*; Table 2), suggesting that many melanogenesis markers could be associated with shorter survival. A possible bias due to a difference in tumour burden was checked using S100B, and data showed that the latter marker did not differ significantly between the two groups (Table 2, Control). The TYRP1 was then chosen to be further evaluated as a new potential marker of poorer prognosis.

### Comparison of probe set signature and TYRP1 mRNA in the training population

In order to determine if TYRP1 mRNA expression genuinely reflected the 278 probe set signature, scores of the signature and TYRP1 were calculated as follows: the score of probe set signature was the mean expression levels (log-transformed values) of upregulated genes minus the mean expression levels (log-transformed values) of downregulated genes, and the score of TYRP1 was the log-transformed value. First, there were significant correlations between the score of the probe set signature and the score of TYRP1 (*ρ*=0.719, *P*<0.001, Spearman's rho). Second, TYRP1 mRNA expression was significantly higher (*P*<0.001, Mann–Whitney test) in the subpopulation of patients with poor prognosis (group 1). Hence, TYRP1 mRNA expression was actually as informative as the probe set signature with respect to patient survival.

### Validation of TYRP1 microarray data by real-time PCR

#### Training population

Microarray data were validated by real-time PCR measurement of TYRP1 mRNA expression in the skin metastases (*N*=13) of the training population. We validated the microarray data on skin metastases only in order to match with the validation population. Results showed a significant correlation between microarray and PCR data (*ρ*=0.780, *P*=0.002, Spearman's rho). Accordingly, TYRP1 mRNA expression (real-time PCR) was significantly higher (*P*=0.001, Mann–Whitney test) in group 1 (OS <30 months) when compared with group 2 (OS ⩾30 months).

#### Validation population

**Figure 1:**
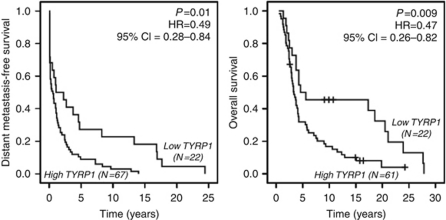
The TYRP1 mRNA expression and survival of 89 patients with melanoma skin metastases. The distant metastasis-free survival and the overall survival curves (Kaplan–Meier analysis) were determined for patients of the validation population. Patients were distributed according to the TYRP1 mRNA levels (real-time PCR determination) as defined in the ‘Results’. Cox regressions were calculated to determine *P-*values, hazard ratios (HRs) and 95% confidence intervals (CIs). The symbol ‘+’ indicates the patients who were alive at the time of analysis. The mRNA expression of TYRP1 had a positive predictive value of 94% (*P*=0.01, Fisher's exact test) for a shorter DMFS (<7.5 years) and a positive predictive value of 96% (*P*<0.001) for a shorter OS (<15 years).

The expression of TYRP1 mRNA was evaluated by real-time PCR in an independent cohort of skin metastases (*N*=89, Table 1, validation population). The population was divided into quartiles on the basis of TYRP1 levels and subgroups were subjected to Kaplan–Meier analysis. The quartile corresponding to the lowest TYRP1 levels presented a better survival when compared with the three other quartiles (higher TYRP1 levels), for which the Kaplan–Meier curves were not significantly different (DMFS *P*=0.221, OS *P*=0.112, Cox regression). Therefore, a cutoff point at the first quartile was set in order to divide the population in two groups of ‘low’ and ‘high’ TYRP1 mRNA levels. Hence, in the validation population, high TYRP1 mRNA expression was significantly associated with a shorter DMFS and a shorter OS (Figure 1). A possible difference in tumour burden between the two groups was checked by real-time PCR of S100B and the analysis did not reveal significant difference (*P*=0.91; Mann–Whitney). Accordingly, we found a very good correlation between TYRP1/*β*-actin and TYRP1/S100B ratios (*ρ*=0.770, *P*<0.001, Spearman's rho), indicating that S100B and *β*-actin changes are closely related, and further supporting no significant difference in tumour load.

### Serial measurements of TYRP1 expression in recurrent skin metastases of the same patient

**Figure 2:**
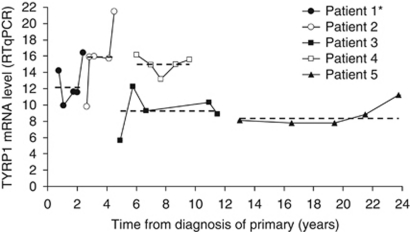
Variation of TYRP1 mRNA expression levels over time in recurrent skin metastases within the same patient. The levels of expression of TYRP1 mRNA were evaluated in quadruplicate by real-time PCR in five different melanoma skin metastases obtained over years from each of five patients. The TYRP1 mRNA levels (median) are plotted against time from diagnosis of primary. Dashed line indicates the median value of TYRP1 mRNA expression for each patient. For each patient, TYRP1 mRNA levels were compared using Kruskal–Wallis test (^★^significant). A weak significant difference was calculated in patient 1 (*P*=0.03).

As the data suggest that TYRP1 mRNA expression in skin metastases has prognostic value regardless of the time of their development (median: 30 months, range: 1–332), it was measured in five consecutive metastases resected from each of five patients (Figure 2). The mRNA levels of TYRP1 remained within a narrow range with a coefficient of variation of <16% for each patient.

### Evaluation of Tyrp1 protein expression in skin metastases

**Figure 3:**
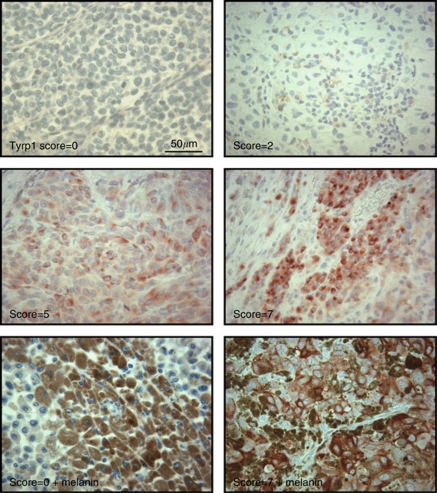
Representative micrographs of Tyrp1 protein expression in paraffin-embedded specimens of melanoma skin metastasis. The expression of Tyrp1 protein was evaluated by immunohistochemistry in 52 amelanotic or pigmented samples. A score from 0 to 8 was calculated by adding a score reflecting the proportion of positively stained cells (none=0; <1/100=1; 1/100 to 1/10=2; 1/10 to 1/3=3; 1/3 to 2/3=4; and >2/3=5) to a score reflecting the staining intensity (none=0; weak=1; intermediate=2; and strong=3). Cytoplasmic red staining was considered as positive. Brown granules represent melanin pigment.

The expression of the protein (Tyrp1/gp75) was examined by immunohistochemistry (IHC) in a panel of paraffin-embedded biopsies from the skin metastases of the validation population (*N*=52; Figure 3). The Tyrp1 protein immunoreactivity was apparent only in 48% of cases positive for TYRP1 mRNA expression. However, in samples with positive staining, there was a significant correlation between staining scores and mRNA levels (real-time PCR) (*ρ*=0.488, *P*=0.034, Spearman's rho).

### Correlation between TYRP1 mRNA or Tyrp1 protein and prognostic parameters at primary

The expressions of TYRP1 mRNA and Tyrp1 protein in skin metastases were correlated with the AJCC prognostic parameters at diagnosis (Table 3). Both TYRP1 mRNA and Tyrp1 protein expressions significantly correlated with Breslow thickness. Nonsignificant trends were recorded between Tyrp1 protein and the ulceration status or the number of positive lymph nodes. Thus, TYRP1 mRNA and Tyrp1 protein expressions in cutaneous melanoma metastases remain associated with prognostic features of the corresponding primary lesions, regardless of the interval between the diagnosis of the primary and the occurrence of the cutaneous metastases.

## Discussion

First, 278 probe sets associated with a short survival of patients with melanoma metastases have been identified by microarray analysis in skin and lymph node metastases. The TYRP1 expression ranked first and, alone, was predictive for DMFS and OS as validated in skin metastases, with the skin being the first site of recurrence (56% of all patients) (Savoia *et al*, 2009). The TYRP1 also significantly correlated with Breslow thickness, which is the most accurate prognostic parameter of the corresponding primary lesion, and thus brings information that is quite different, although complementary, from the ‘mitotic index’ that is calculated at the time of metastasis itself (Bogunovic *et al*, 2009).

We ruled out any possible bias due to variation in tumour burden between the compared groups in both the training and validation populations by analysing the specific melanocyte marker S100B; the latter showed no difference in expression. Moreover, as *TYRP1* gene is related to pigmentation and is exclusively expressed in melanocytes and melanoma cells, the documented changes in its expression is restricted to tumour tissues.

Corroborating our results, a previous study reported that the repression of TYRP1 is concomitant to the induction of an isoform of the microtubule-associated protein 2 (MAP-2), a marker of immature neurons (Fang *et al*, 2001), and that patients with MAP2-positive (hence, low TYRP1) primary melanomas have a significantly improved survival (Soltani *et al*, 2005).

Importantly, TYRP1 sorted together with five other melanogenesis-related genes (*SILV*, *DCT*, *OCA2*, *TYR* and *MITF*), which were also contained in the probe set signature, point to an active pigmentation process. In this context, a previous study using cDNA microarray analysis showed that high expression of DCT/TYRP2 in metastatic melanoma was associated with a shorter patient survival (Mandruzzato *et al*, 2006). The question is why would a melanocyte differentiation marker, such as TYRP1, be associated with survival? The hallmark of differentiated melanocytes is an active pigmentation process that may increase with tumour burden. In addition, as melanoma cells are coupling proliferation and differentiation through MITF (Wellbrock *et al*, 2008), the transcription factor that control the synthesis of *TYR*, *TYRP1* and *DCT* genes (Murisier and Beermann, 2006), we may reasonably assume that high level of expression of genes involved in melanogenesis results from a net balance of the MITF activity.

Besides the possible role of human Tyrp1 as a melanogenic enzyme (Hearing *et al*, 1992), several observations provide evidence supporting its putative implication in cell survival. First, Tyrp1 mutation or decrease in expression interferes with melanosome maturation in mouse melanocytes and, most interestingly, attenuates cell proliferation, without evidence of necrosis or apoptosis (Sarangarajan *et al*, 2000). Second, both Tyrp1 and Dct/Tyrp2 protect the melanocyte against the cytotoxicity of toxic melanin intermediates produced by tyrosinase, without affecting tyrosinase expression or its activity (Rad *et al*, 2004).

The discrepancies between TYRP1 mRNA levels (frozen tissue samples) and Tyrp1/gp75 protein expression (corresponding paraffin embedded samples) that we found in skin metastases of about half of the patients, including some with high TYRP1 mRNA expression but no protein if any, may suggest possible post-transcriptional and/or post-translational events altering the protein recognition by the antibody despite the best choice of the G3E6 antibody that recognises the unglycosylated C-terminus of the protein. Our finding of this discrepancy as well as the use of various anti-Tyrp1 antibodies in previous studies could explain the previously reported lack of association between Tyrp1 protein expression and disease-free or OS (Bolander *et al*, 2008), and the absence of Tyrp1 protein in the vertical/invasive growth phase of primary lesion (Fang *et al*, 2001). Furthermore, no or low expression of Tyrp1 protein might be explained by (1) the absence of a putative Tyrp1 chaperone, such as calnexin, which is required to stabilise the protein (Jimbow *et al*, 1997), (2) a defect in PI3K-dependent Tyrp1 maturation and trafficking to melanosomes (Chen *et al*, 2001), and/or (3) a loss of Rab proteins, such as Rab38/32, which are involved in the stability of melanogenic enzymes (Wasmeier *et al*, 2006).

On the other hand, as the activation of melanogenesis enzymes may lead to visible pigmentation, the latter was checked in the same IHC slides, but no correlation was found with Tyrp1 protein expression (example in Figure 3). This finding is consistent with previous studies showing that Tyrp1 can be regulated independently of tyrosinase and pigmentation in mature melanocytes (Vijayasaradhi *et al*, 1995) and that tyrosinase mRNA or protein expression does not always correlate with pigmentation (Watabe *et al*, 2004).

Finally, our findings of an association between high TYRP1/Tyrp1 levels and shorter patient survival, together with a conserved TYRP1 expression throughout the course of the disease, are in line with previous clinical studies that identified circulating anti-Tyrp1 autoantibodies in melanoma patients (Mattes *et al*, 1983) and that demonstrated melanoma rejection and higher survival in mice treated with a mouse monoclonal antibody against Tyrp1 (Takechi *et al*, 1996), and support the recent clinical development of a new human anti-Tyrp1 monoclonal antibody for melanoma immunotherapy (Patel *et al*, 2007) (http://clinicaltrials.gov/ct2/show/NCT01137006).

In conclusion, we found that TYRP1 gene expression level in melanoma skin metastases correlates with both DMFS and OS and with Breslow thickness. Thus, TYRP1 could emerge as a valuable prognostic marker, especially in melanoma patients where important prognostic factors at diagnosis cannot be evaluated (namely unknown or ulcerated primaries) and in metastases of thin melanomas. Our observation of a fairly conserved TYRP1 expression during disease progression further supports its use as a target for antimelanoma therapy.

## Figures and Tables

**Table 1:** Characteristics of metastasis samples from melanoma patients

	**Training population**	**Validation population**
Number of samples	32	89
*Type of metastasis*		
Skin	13	89
Lymph node	19	0
		
*Gender*		
Male	15	35
Female	17	54
		
Median age (range), years^a^	49 (26–88)	58 (20–88)
Median Breslow (range), mm^b^	2.0 (0.9–9.1)	3.2 (0.3–15)
Median DMFS (range), months	14 (0–105)	9 (0–293)
Median OS (range), months	49 (8–186)	45 (6–334)

Abbreviations: DMFS=distant metastasis-free survival; OS=overall survival.

a Age at the diagnosis of primary melanoma.

b Breslow is the thickness of primary tumours as determined by histopathological examination.

**Table 2:** Ranking of the pigmentation-related genes included in the 278 probe set signature associated with a poorer survival

**Rank^a^**	***P*-value^b^**	**FDR**	**Geom. mean of intensities in OS <30 months**	**Geom. mean of intensities in OS >30 months**	**Fold change**	**Probe set**	**Gene symbol**	**Description**
1	0.00004	0.15	687.4	20.3	33.9	205694_at	*TYRP1*	*Tyrosinase-related protein 1*
2	0.00040	0.19	3077.2	186.7	16.5	209848_s_at	*SILV*	*Silver homolog (mouse)*
4	0.00103	0.20	1291.6	97.0	13.3	205338_s_at	*DCT*	*Dopachrome tautomerase (tyrosine-related protein 2)*
13	0.00174	0.21	145.3	18.0	8.1	206498_at	*OCA2*	*Oculocutaneous albinism II*
15	0.02823	0.33	3170.1	496.5	6.4	206630_at	*TYR*	*Tyrosinase (oculo-cutaneous albinism IA)*
99	0.02147	0.32	1054.3	375.4	2.8	207233_s_at	*MITF*	*Microphthalmia-associated transcription factor*
Control^c^	0.649	—	929.0	1145.2	0.8	209686_at	*S100B*	*S100 calcium binding protein B*

Abbreviations: Geom=geometric; FDR=false discovery rate; OS=overall survival.

a Position in the complete list (278 probe sets).

b Two-sample *t*-test.

c Assessed as control for difference in tumour load.

**Table 3:** Association of TYRP1/Tyrp1 mRNA/protein expression with pathological parameters of primaries

	**TYRP1 mRNA**	**Tyrp1 protein**
*Breslow* a		
*N*	87	41
*ρ*	0.281	0.383
*P*	**0.008**	**0.013**
*Lymph nodes*^a^,^b^		
*N*	88	45
*ρ*	0.095	0.289
*P*	0.377	0.054
*Ulceration (no/yes)* c		
*N*	87	41
*P*	0.368	0.056

Abbreviation: *TYRP1*=*tyrosinase-related protein 1*.

a Correlation test (Spearman's rho).

b Number of positive lymph nodes at primary.

c Nonparametric test (Mann–Whitney). Bold numbers indicate significant *P*-values.
