# Supplementary material for: TYRP1 mRNA expression in melanoma metastases correlates with clinical outcome
Source: Br J Cancer. 2011 Nov 1;105(11):1726–32. doi: 10.1038/bjc.2011.451 (PMC3242608; doi:10.1038/bjc.2011.451)
Supplement: Supplementary Table 1 [file bjc2011451x1.doc]

**Supplementary Table 1:** Ranking of the 278 probe sets associated with a poorer survival

Class comparison between groups of arrays based on patient survival (group 1 (N=10): OS < 30 months; group 2 (N=22): OS > 30 months)

Type of univariate test used: two-sample T-test

Nominal significance level of each univariate test: 0.05

Fold-change strictly higher than 2.5

278 probe sets sorted

Presentation by descending fold-change

Genes related to melanogenesis are highlighted

| **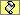Rank** | **p-value** | **FDR**  **(**False Discovery Rate) | **Geom mean of intensities in OS<30 months** | **Geom mean of intensities in OS>30 months** | **Fold-change** | **Probe set** | **Gene symbol** | **Description** |
| --- | --- | --- | --- | --- | --- | --- | --- | --- |
| 1 | 0.00004 | 0.15 | 687.45 | 20.27 | 33.92 | [205694_at](https://www.affymetrix.com/LinkServlet?probeset=205694_at) | [TYRP1](http://www.ncbi.nlm.nih.gov/entrez/query.fcgi?cmd=search&db=gene&term=TYRP1) | tyrosinase-related protein 1 |
| 2 | 0.00040 | 0.19 | 3077.20 | 186.70 | 16.48 | [209848_s_at](https://www.affymetrix.com/LinkServlet?probeset=209848_s_at) | [SILV](http://www.ncbi.nlm.nih.gov/entrez/query.fcgi?cmd=search&db=gene&term=SILV) | silver homolog (mouse) |
| 3 | 0.00066 | 0.20 | 1016.10 | 65.43 | 15.53 | [240386_at](https://www.affymetrix.com/LinkServlet?probeset=240386_at) | [TRPM1](http://www.ncbi.nlm.nih.gov/entrez/query.fcgi?cmd=search&db=gene&term=TRPM1) | transient receptor potential cation channel, subfamily M, member 1 |
| 4 | 0.00103 | 0.20 | 1291.64 | 97.00 | 13.32 | [205338_s_at](https://www.affymetrix.com/LinkServlet?probeset=205338_s_at) | [DCT](http://www.ncbi.nlm.nih.gov/entrez/query.fcgi?cmd=search&db=gene&term=DCT) | dopachrome tautomerase (dopachrome delta-isomerase, tyrosine-related protein 2) |
| 5 | 0.00169 | 0.20 | 1042.34 | 83.98 | 12.41 | [237070_at](https://www.affymetrix.com/LinkServlet?probeset=237070_at) | [TRPM1](http://www.ncbi.nlm.nih.gov/entrez/query.fcgi?cmd=search&db=gene&term=TRPM1) | transient receptor potential cation channel, subfamily M, member 1 |
| 6 | 0.00013 | 0.16 | 499.29 | 46.17 | 10.81 | [232122_s_at](https://www.affymetrix.com/LinkServlet?probeset=232122_s_at) | [VEPH1](http://www.ncbi.nlm.nih.gov/entrez/query.fcgi?cmd=search&db=gene&term=VEPH1) | ventricular zone expressed PH domain homolog 1 (zebrafish) |
| 7 | 0.00346 | 0.24 | 569.46 | 60.12 | 9.47 | [216512_s_at](https://www.affymetrix.com/LinkServlet?probeset=216512_s_at) | [DCT](http://www.ncbi.nlm.nih.gov/entrez/query.fcgi?cmd=search&db=gene&term=DCT) | dopachrome tautomerase (dopachrome delta-isomerase, tyrosine-related protein 2) |
| 8 | 0.00461 | 0.24 | 404.83 | 45.32 | 8.93 | [243167_at](https://www.affymetrix.com/LinkServlet?probeset=243167_at) | [ABCB5](http://www.ncbi.nlm.nih.gov/entrez/query.fcgi?cmd=search&db=gene&term=ABCB5) | ATP-binding cassette, sub-family B (MDR/TAP), member 5 |
| 9 | 0.00578 | 0.25 | 234.42 | 26.30 | 8.91 | [240717_at](https://www.affymetrix.com/LinkServlet?probeset=240717_at) | [ABCB5](http://www.ncbi.nlm.nih.gov/entrez/query.fcgi?cmd=search&db=gene&term=ABCB5) | ATP-binding cassette, sub-family B (MDR/TAP), member 5 |
| 10 | 0.00658 | 0.26 | 1415.85 | 165.87 | 8.54 | [206696_at](https://www.affymetrix.com/LinkServlet?probeset=206696_at) | [GPR143](http://www.ncbi.nlm.nih.gov/entrez/query.fcgi?cmd=search&db=gene&term=GPR143) | G protein-coupled receptor 143 |
| 11 | 0.00694 | 0.26 | 764.88 | 92.25 | 8.29 | [205337_at](https://www.affymetrix.com/LinkServlet?probeset=205337_at) | [DCT](http://www.ncbi.nlm.nih.gov/entrez/query.fcgi?cmd=search&db=gene&term=DCT) | dopachrome tautomerase (dopachrome delta-isomerase, tyrosine-related protein 2) |
| 12 | 0.00098 | 0.20 | 253.26 | 30.76 | 8.23 | [237069_s_at](https://www.affymetrix.com/LinkServlet?probeset=237069_s_at) | [TRPM1](http://www.ncbi.nlm.nih.gov/entrez/query.fcgi?cmd=search&db=gene&term=TRPM1) | transient receptor potential cation channel, subfamily M, member 1 |
| 13 | 0.00174 | 0.21 | 145.28 | 18.04 | 8.05 | [206498_at](https://www.affymetrix.com/LinkServlet?probeset=206498_at) | [OCA2](http://www.ncbi.nlm.nih.gov/entrez/query.fcgi?cmd=search&db=gene&term=OCA2) | oculocutaneous albinism II |
| 14 | 0.00071 | 0.20 | 173.10 | 26.05 | 6.64 | [212664_at](https://www.affymetrix.com/LinkServlet?probeset=212664_at) | [TUBB4](http://www.ncbi.nlm.nih.gov/entrez/query.fcgi?cmd=search&db=gene&term=TUBB4) | tubulin, beta 4 |
| 15 | 0.02823 | 0.33 | 3170.06 | 496.50 | 6.38 | [206630_at](https://www.affymetrix.com/LinkServlet?probeset=206630_at) | [TYR](http://www.ncbi.nlm.nih.gov/entrez/query.fcgi?cmd=search&db=gene&term=TYR) | tyrosinase (oculocutaneous albinism IA) |
| 16 | 0.00449 | 0.24 | 213.64 | 34.53 | 6.19 | [206479_at](https://www.affymetrix.com/LinkServlet?probeset=206479_at) | [TRPM1](http://www.ncbi.nlm.nih.gov/entrez/query.fcgi?cmd=search&db=gene&term=TRPM1) | transient receptor potential cation channel, subfamily M, member 1 |
| 17 | 0.00014 | 0.17 | 116.08 | 18.80 | 6.17 | [230251_at](https://www.affymetrix.com/LinkServlet?probeset=230251_at) | [C6orf176](http://www.ncbi.nlm.nih.gov/entrez/query.fcgi?cmd=search&db=gene&term=C6orf176) | chromosome 6 open reading frame 176 |
| 18 | 0.02280 | 0.32 | 1580.00 | 260.54 | 6.06 | [206426_at](https://www.affymetrix.com/LinkServlet?probeset=206426_at) | [MLANA](http://www.ncbi.nlm.nih.gov/entrez/query.fcgi?cmd=search&db=gene&term=MLANA) | melan-A |
| 19 | 0.00001 | 0.15 | 83.62 | 14.11 | 5.93 | [227048_at](https://www.affymetrix.com/LinkServlet?probeset=227048_at) | [LAMA1](http://www.ncbi.nlm.nih.gov/entrez/query.fcgi?cmd=search&db=gene&term=LAMA1) | laminin, alpha 1 |
| 20 | 0.00050 | 0.19 | 55.21 | 9.67 | 5.71 | [221623_at](https://www.affymetrix.com/LinkServlet?probeset=221623_at) | [BCAN](http://www.ncbi.nlm.nih.gov/entrez/query.fcgi?cmd=search&db=gene&term=BCAN) | brevican |
| 21 | 0.03341 | 0.34 | 3673.63 | 647.06 | 5.68 | [206427_s_at](https://www.affymetrix.com/LinkServlet?probeset=206427_s_at) | [MLANA](http://www.ncbi.nlm.nih.gov/entrez/query.fcgi?cmd=search&db=gene&term=MLANA) | melan-A |
| 22 | 0.00059 | 0.20 | 553.94 | 99.98 | 5.54 | [205051_s_at](https://www.affymetrix.com/LinkServlet?probeset=205051_s_at) | [KIT](http://www.ncbi.nlm.nih.gov/entrez/query.fcgi?cmd=search&db=gene&term=KIT) | v-kit Hardy-Zuckerman 4 feline sarcoma viral oncogene homolog |
| 23 | 0.00701 | 0.26 | 186.10 | 33.81 | 5.50 | [216513_at](https://www.affymetrix.com/LinkServlet?probeset=216513_at) | [DCT](http://www.ncbi.nlm.nih.gov/entrez/query.fcgi?cmd=search&db=gene&term=DCT) | dopachrome tautomerase (dopachrome delta-isomerase, tyrosine-related protein 2) |
| 24 | 0.00818 | 0.27 | 405.23 | 74.31 | 5.45 | [220245_at](https://www.affymetrix.com/LinkServlet?probeset=220245_at) | [SLC45A2](http://www.ncbi.nlm.nih.gov/entrez/query.fcgi?cmd=search&db=gene&term=SLC45A2) | solute carrier family 45, member 2 |
| 25 | 0.01746 | 0.30 | 679.67 | 126.40 | 5.38 | [1555505_a_at](https://www.affymetrix.com/LinkServlet?probeset=1555505_a_at) | [TYR](http://www.ncbi.nlm.nih.gov/entrez/query.fcgi?cmd=search&db=gene&term=TYR) | tyrosinase (oculocutaneous albinism IA) |
| 26 | 0.00093 | 0.20 | 429.73 | 82.34 | 5.22 | [215028_at](https://www.affymetrix.com/LinkServlet?probeset=215028_at) | [SEMA6A](http://www.ncbi.nlm.nih.gov/entrez/query.fcgi?cmd=search&db=gene&term=SEMA6A) | sema domain, transmembrane domain (TM), and cytoplasmic domain, (semaphorin) 6A |
| 27 | 0.01215 | 0.29 | 397.90 | 77.90 | 5.11 | [209569_x_at](https://www.affymetrix.com/LinkServlet?probeset=209569_x_at) | [D4S234E](http://www.ncbi.nlm.nih.gov/entrez/query.fcgi?cmd=search&db=gene&term=D4S234E) | DNA segment on chromosome 4 (unique) 234 expressed sequence |
| 28 | 0.00426 | 0.24 | 300.46 | 59.50 | 5.05 | [209072_at](https://www.affymetrix.com/LinkServlet?probeset=209072_at) | [MBP](http://www.ncbi.nlm.nih.gov/entrez/query.fcgi?cmd=search&db=gene&term=MBP) | myelin basic protein |
| 29 | 0.00377 | 0.24 | 120.30 | 23.87 | 5.04 | [221644_s_at](https://www.affymetrix.com/LinkServlet?probeset=221644_s_at) | [SLC45A2](http://www.ncbi.nlm.nih.gov/entrez/query.fcgi?cmd=search&db=gene&term=SLC45A2) | solute carrier family 45, member 2 |
| 30 | 0.02463 | 0.32 | 357.24 | 73.24 | 4.88 | [1555504_at](https://www.affymetrix.com/LinkServlet?probeset=1555504_at) | [TYR](http://www.ncbi.nlm.nih.gov/entrez/query.fcgi?cmd=search&db=gene&term=TYR) | tyrosinase (oculocutaneous albinism IA) |
| 31 | 0.00367 | 0.24 | 306.19 | 65.95 | 4.64 | [236972_at](https://www.affymetrix.com/LinkServlet?probeset=236972_at) | [TRIM63](http://www.ncbi.nlm.nih.gov/entrez/query.fcgi?cmd=search&db=gene&term=TRIM63) | tripartite motif-containing 63 |
| 32 | 0.01718 | 0.30 | 82.25 | 17.77 | 4.63 | [1569072_s_at](https://www.affymetrix.com/LinkServlet?probeset=1569072_s_at) | [ABCB5](http://www.ncbi.nlm.nih.gov/entrez/query.fcgi?cmd=search&db=gene&term=ABCB5) | ATP-binding cassette, sub-family B (MDR/TAP), member 5 |
| 33 | 0.02703 | 0.33 | 314.76 | 68.97 | 4.56 | [225846_at](https://www.affymetrix.com/LinkServlet?probeset=225846_at) | [RBM35A](http://www.ncbi.nlm.nih.gov/entrez/query.fcgi?cmd=search&db=gene&term=RBM35A) | RNA binding motif protein 35A |
| 34 | 0.01276 | 0.29 | 131.42 | 29.48 | 4.46 | [219464_at](https://www.affymetrix.com/LinkServlet?probeset=219464_at) | [CA14](http://www.ncbi.nlm.nih.gov/entrez/query.fcgi?cmd=search&db=gene&term=CA14) | carbonic anhydrase XIV |
| 35 | 0.00019 | 0.18 | 86.58 | 19.42 | 4.46 | [225660_at](https://www.affymetrix.com/LinkServlet?probeset=225660_at) | [SEMA6A](http://www.ncbi.nlm.nih.gov/entrez/query.fcgi?cmd=search&db=gene&term=SEMA6A) | sema domain, transmembrane domain (TM), and cytoplasmic domain, (semaphorin) 6A |
| 36 | 0.01149 | 0.28 | 214.64 | 52.45 | 4.09 | [219195_at](https://www.affymetrix.com/LinkServlet?probeset=219195_at) | [PPARGC1A](http://www.ncbi.nlm.nih.gov/entrez/query.fcgi?cmd=search&db=gene&term=PPARGC1A) | peroxisome proliferator-activated receptor gamma, coactivator 1 alpha |
| 37 | 0.00040 | 0.19 | 58.96 | 14.56 | 4.05 | [226766_at](https://www.affymetrix.com/LinkServlet?probeset=226766_at) | [ROBO2](http://www.ncbi.nlm.nih.gov/entrez/query.fcgi?cmd=search&db=gene&term=ROBO2) | roundabout, axon guidance receptor, homolog 2 (Drosophila) |
| 38 | 0.00040 | 0.19 | 130.17 | 32.47 | 4.01 | [1552256_a_at](https://www.affymetrix.com/LinkServlet?probeset=1552256_a_at) | [SCARB1](http://www.ncbi.nlm.nih.gov/entrez/query.fcgi?cmd=search&db=gene&term=SCARB1) | scavenger receptor class B, member 1 |
| 39 | 0.02459 | 0.32 | 158.94 | 39.95 | 3.98 | [203108_at](https://www.affymetrix.com/LinkServlet?probeset=203108_at) | [GPRC5A](http://www.ncbi.nlm.nih.gov/entrez/query.fcgi?cmd=search&db=gene&term=GPRC5A) | G protein-coupled receptor, family C, group 5, member A |
| 40 | 0.00314 | 0.23 | 27.65 | 6.98 | 3.96 | [206913_at](https://www.affymetrix.com/LinkServlet?probeset=206913_at) | [BAAT](http://www.ncbi.nlm.nih.gov/entrez/query.fcgi?cmd=search&db=gene&term=BAAT) | bile acid Coenzyme A: amino acid N-acyltransferase (glycine N-choloyltransferase) |
| 41 | 0.00485 | 0.24 | 551.66 | 142.77 | 3.86 | [209959_at](https://www.affymetrix.com/LinkServlet?probeset=209959_at) | [NR4A3](http://www.ncbi.nlm.nih.gov/entrez/query.fcgi?cmd=search&db=gene&term=NR4A3) | nuclear receptor subfamily 4, group A, member 3 |
| 42 | 0.00477 | 0.24 | 203.61 | 52.77 | 3.86 | [204187_at](https://www.affymetrix.com/LinkServlet?probeset=204187_at) | [GMPR](http://www.ncbi.nlm.nih.gov/entrez/query.fcgi?cmd=search&db=gene&term=GMPR) | guanosine monophosphate reductase |
| 43 | 0.00920 | 0.27 | 232.88 | 60.46 | 3.85 | [238621_at](https://www.affymetrix.com/LinkServlet?probeset=238621_at) | [FMN1](http://www.ncbi.nlm.nih.gov/entrez/query.fcgi?cmd=search&db=gene&term=FMN1) | formin 1 |
| 44 | 0.00019 | 0.18 | 36.66 | 9.56 | 3.83 | [219107_at](https://www.affymetrix.com/LinkServlet?probeset=219107_at) | [BCAN](http://www.ncbi.nlm.nih.gov/entrez/query.fcgi?cmd=search&db=gene&term=BCAN) | brevican |
| 45 | 0.01784 | 0.30 | 74.67 | 19.52 | 3.82 | [1556410_a_at](https://www.affymetrix.com/LinkServlet?probeset=1556410_a_at) | [KRTAP19-1](http://www.ncbi.nlm.nih.gov/entrez/query.fcgi?cmd=search&db=gene&term=KRTAP19-1) | keratin associated protein 19-1 |
| 46 | 0.00539 | 0.25 | 133.22 | 35.26 | 3.78 | [204654_s_at](https://www.affymetrix.com/LinkServlet?probeset=204654_s_at) | [TFAP2A](http://www.ncbi.nlm.nih.gov/entrez/query.fcgi?cmd=search&db=gene&term=TFAP2A) | transcription factor AP-2 alpha (activating enhancer binding protein 2 alpha) |
| 47 | 0.01784 | 0.30 | 154.31 | 41.11 | 3.75 | [204777_s_at](https://www.affymetrix.com/LinkServlet?probeset=204777_s_at) | [MAL](http://www.ncbi.nlm.nih.gov/entrez/query.fcgi?cmd=search&db=gene&term=MAL) | mal, T-cell differentiation protein |
| 48 | 0.04175 | 0.37 | 163.58 | 44.24 | 3.70 | [235400_at](https://www.affymetrix.com/LinkServlet?probeset=235400_at) | [FCRLA](http://www.ncbi.nlm.nih.gov/entrez/query.fcgi?cmd=search&db=gene&term=FCRLA) | Fc receptor-like A |
| 49 | 0.00002 | 0.15 | 62.86 | 17.00 | 3.70 | [235961_at](https://www.affymetrix.com/LinkServlet?probeset=235961_at) | [GPR161](http://www.ncbi.nlm.nih.gov/entrez/query.fcgi?cmd=search&db=gene&term=GPR161) | G protein-coupled receptor 161 |
| 50 | 0.00064 | 0.20 | 2214.52 | 600.01 | 3.69 | [223449_at](https://www.affymetrix.com/LinkServlet?probeset=223449_at) | [SEMA6A](http://www.ncbi.nlm.nih.gov/entrez/query.fcgi?cmd=search&db=gene&term=SEMA6A) | sema domain, transmembrane domain (TM), and cytoplasmic domain, (semaphorin) 6A |
| 51 | 0.00129 | 0.20 | 55.06 | 15.05 | 3.66 | [203911_at](https://www.affymetrix.com/LinkServlet?probeset=203911_at) | [RAP1GAP](http://www.ncbi.nlm.nih.gov/entrez/query.fcgi?cmd=search&db=gene&term=RAP1GAP) | RAP1 GTPase activating protein |
| 52 | 0.01634 | 0.30 | 211.46 | 58.19 | 3.63 | [204857_at](https://www.affymetrix.com/LinkServlet?probeset=204857_at) | [MAD1L1](http://www.ncbi.nlm.nih.gov/entrez/query.fcgi?cmd=search&db=gene&term=MAD1L1) | MAD1 mitotic arrest deficient-like 1 (yeast) |
| 53 | 0.02175 | 0.32 | 117.02 | 32.54 | 3.60 | [219121_s_at](https://www.affymetrix.com/LinkServlet?probeset=219121_s_at) | [RBM35A](http://www.ncbi.nlm.nih.gov/entrez/query.fcgi?cmd=search&db=gene&term=RBM35A) | RNA binding motif protein 35A |
| 54 | 0.00161 | 0.20 | 114.70 | 31.92 | 3.59 | [1554486_a_at](https://www.affymetrix.com/LinkServlet?probeset=1554486_a_at) | [C6orf114](http://www.ncbi.nlm.nih.gov/entrez/query.fcgi?cmd=search&db=gene&term=C6orf114) | chromosome 6 open reading frame 114 |
| 55 | 0.00221 | 0.22 | 178.30 | 49.85 | 3.58 | [223795_at](https://www.affymetrix.com/LinkServlet?probeset=223795_at) | [TSPAN10](http://www.ncbi.nlm.nih.gov/entrez/query.fcgi?cmd=search&db=gene&term=TSPAN10) | tetraspanin 10 |
| 56 | 0.00334 | 0.24 | 1419.94 | 399.64 | 3.55 | [205174_s_at](https://www.affymetrix.com/LinkServlet?probeset=205174_s_at) | [QPCT](http://www.ncbi.nlm.nih.gov/entrez/query.fcgi?cmd=search&db=gene&term=QPCT) | glutaminyl-peptide cyclotransferase |
| 57 | 0.03937 | 0.36 | 230.12 | 65.81 | 3.50 | [221577_x_at](https://www.affymetrix.com/LinkServlet?probeset=221577_x_at) | [GDF15](http://www.ncbi.nlm.nih.gov/entrez/query.fcgi?cmd=search&db=gene&term=GDF15) | growth differentiation factor 15 |
| 58 | 0.00862 | 0.27 | 304.09 | 87.16 | 3.49 | [1554026_a_at](https://www.affymetrix.com/LinkServlet?probeset=1554026_a_at) | [MYO10](http://www.ncbi.nlm.nih.gov/entrez/query.fcgi?cmd=search&db=gene&term=MYO10) | myosin X |
| 59 | 0.02595 | 0.33 | 823.35 | 239.22 | 3.44 | [209570_s_at](https://www.affymetrix.com/LinkServlet?probeset=209570_s_at) | [D4S234E](http://www.ncbi.nlm.nih.gov/entrez/query.fcgi?cmd=search&db=gene&term=D4S234E) | DNA segment on chromosome 4 (unique) 234 expressed sequence |
| 60 | 0.00023 | 0.19 | 83.64 | 24.30 | 3.44 | [235213_at](https://www.affymetrix.com/LinkServlet?probeset=235213_at) | [ITPKB](http://www.ncbi.nlm.nih.gov/entrez/query.fcgi?cmd=search&db=gene&term=ITPKB) | inositol 1,4,5-trisphosphate 3-kinase B |
| 61 | 0.01746 | 0.30 | 78.31 | 22.76 | 3.44 | [235182_at](https://www.affymetrix.com/LinkServlet?probeset=235182_at) | [C20orf82](http://www.ncbi.nlm.nih.gov/entrez/query.fcgi?cmd=search&db=gene&term=C20orf82) | chromosome 20 open reading frame 82 |
| 62 | 0.03230 | 0.34 | 98.80 | 28.84 | 3.43 | [209616_s_at](https://www.affymetrix.com/LinkServlet?probeset=209616_s_at) | [CES1](http://www.ncbi.nlm.nih.gov/entrez/query.fcgi?cmd=search&db=gene&term=CES1) | carboxylesterase 1 (monocyte/macrophage serine esterase 1) |
| 63 | 0.00073 | 0.20 | 121.87 | 35.58 | 3.42 | [201819_at](https://www.affymetrix.com/LinkServlet?probeset=201819_at) | [SCARB1](http://www.ncbi.nlm.nih.gov/entrez/query.fcgi?cmd=search&db=gene&term=SCARB1) | scavenger receptor class B, member 1 |
| 64 | 0.02749 | 0.33 | 700.40 | 205.34 | 3.41 | [203510_at](https://www.affymetrix.com/LinkServlet?probeset=203510_at) | [MET](http://www.ncbi.nlm.nih.gov/entrez/query.fcgi?cmd=search&db=gene&term=MET) | met proto-oncogene (hepatocyte growth factor receptor) |
| 65 | 0.04761 | 0.38 | 400.21 | 118.40 | 3.38 | [201621_at](https://www.affymetrix.com/LinkServlet?probeset=201621_at) | [NBL1](http://www.ncbi.nlm.nih.gov/entrez/query.fcgi?cmd=search&db=gene&term=NBL1) | neuroblastoma, suppression of tumorigenicity 1 |
| 66 | 0.00857 | 0.27 | 294.81 | 87.22 | 3.38 | [202017_at](https://www.affymetrix.com/LinkServlet?probeset=202017_at) | [EPHX1](http://www.ncbi.nlm.nih.gov/entrez/query.fcgi?cmd=search&db=gene&term=EPHX1) | epoxide hydrolase 1, microsomal (xenobiotic) |
| 67 | 0.04003 | 0.36 | 670.08 | 200.70 | 3.34 | [37005_at](https://www.affymetrix.com/LinkServlet?probeset=37005_at) | [NBL1](http://www.ncbi.nlm.nih.gov/entrez/query.fcgi?cmd=search&db=gene&term=NBL1) | neuroblastoma, suppression of tumorigenicity 1 |
| 68 | 0.01220 | 0.29 | 134.83 | 40.41 | 3.34 | [227933_at](https://www.affymetrix.com/LinkServlet?probeset=227933_at) | [LINGO1](http://www.ncbi.nlm.nih.gov/entrez/query.fcgi?cmd=search&db=gene&term=LINGO1) | leucine rich repeat and Ig domain containing 1 |
| 69 | 0.02337 | 0.32 | 92.00 | 27.68 | 3.32 | [207038_at](https://www.affymetrix.com/LinkServlet?probeset=207038_at) | [SLC16A6](http://www.ncbi.nlm.nih.gov/entrez/query.fcgi?cmd=search&db=gene&term=SLC16A6) | solute carrier family 16, member 6 (monocarboxylic acid transporter 7) |
| 70 | 0.00308 | 0.23 | 1161.41 | 351.55 | 3.30 | [201195_s_at](https://www.affymetrix.com/LinkServlet?probeset=201195_s_at) | [SLC7A5](http://www.ncbi.nlm.nih.gov/entrez/query.fcgi?cmd=search&db=gene&term=SLC7A5) | solute carrier family 7 (cationic amino acid transporter, y+ system), member 5 |
| 71 | 0.00311 | 0.23 | 176.56 | 53.50 | 3.30 | [214104_at](https://www.affymetrix.com/LinkServlet?probeset=214104_at) | [GPR161](http://www.ncbi.nlm.nih.gov/entrez/query.fcgi?cmd=search&db=gene&term=GPR161) | G protein-coupled receptor 161 |
| 72 | 0.04024 | 0.36 | 645.03 | 198.05 | 3.26 | [229868_s_at](https://www.affymetrix.com/LinkServlet?probeset=229868_s_at) | [GDF15](http://www.ncbi.nlm.nih.gov/entrez/query.fcgi?cmd=search&db=gene&term=GDF15) | growth differentiation factor 15 |
| 73 | 0.00130 | 0.20 | 100.90 | 31.19 | 3.24 | [223754_at](https://www.affymetrix.com/LinkServlet?probeset=223754_at) | [MGC13057](http://www.ncbi.nlm.nih.gov/entrez/query.fcgi?cmd=search&db=gene&term=MGC13057) | hypothetical protein MGC13057 |
| 74 | 0.01191 | 0.29 | 112.47 | 34.87 | 3.23 | [230962_at](https://www.affymetrix.com/LinkServlet?probeset=230962_at) | [DCLK1](http://www.ncbi.nlm.nih.gov/entrez/query.fcgi?cmd=search&db=gene&term=DCLK1) | doublecortin-like kinase 1 |
| 75 | 0.01052 | 0.28 | 532.43 | 166.83 | 3.19 | [225532_at](https://www.affymetrix.com/LinkServlet?probeset=225532_at) | [CABLES1](http://www.ncbi.nlm.nih.gov/entrez/query.fcgi?cmd=search&db=gene&term=CABLES1) | Cdk5 and Abl enzyme substrate 1 |
| 76 | 0.01620 | 0.30 | 1031.44 | 324.88 | 3.17 | [219412_at](https://www.affymetrix.com/LinkServlet?probeset=219412_at) | [RAB38](http://www.ncbi.nlm.nih.gov/entrez/query.fcgi?cmd=search&db=gene&term=RAB38) | RAB38, member RAS oncogene family |
| 77 | 0.00230 | 0.22 | 65.96 | 21.13 | 3.12 | [226446_at](https://www.affymetrix.com/LinkServlet?probeset=226446_at) | [HES6](http://www.ncbi.nlm.nih.gov/entrez/query.fcgi?cmd=search&db=gene&term=HES6) | hairy and enhancer of split 6 (Drosophila) |
| 78 | 0.00209 | 0.22 | 345.29 | 113.32 | 3.05 | [223741_s_at](https://www.affymetrix.com/LinkServlet?probeset=223741_s_at) | [TTYH2](http://www.ncbi.nlm.nih.gov/entrez/query.fcgi?cmd=search&db=gene&term=TTYH2) | tweety homolog 2 (Drosophila) |
| 79 | 0.01176 | 0.28 | 44.09 | 14.60 | 3.02 | [1554062_at](https://www.affymetrix.com/LinkServlet?probeset=1554062_at) | [XG](http://www.ncbi.nlm.nih.gov/entrez/query.fcgi?cmd=search&db=gene&term=XG) | Xg blood group |
| 80 | 0.01462 | 0.29 | 459.61 | 152.24 | 3.02 | [214255_at](https://www.affymetrix.com/LinkServlet?probeset=214255_at) | [ATP10A](http://www.ncbi.nlm.nih.gov/entrez/query.fcgi?cmd=search&db=gene&term=ATP10A) | ATPase, class V, type 10A |
| 81 | 0.00007 | 0.15 | 121.35 | 40.58 | 2.99 | [1559399_s_at](https://www.affymetrix.com/LinkServlet?probeset=1559399_s_at) | [ZCCHC10](http://www.ncbi.nlm.nih.gov/entrez/query.fcgi?cmd=search&db=gene&term=ZCCHC10) | zinc finger, CCHC domain containing 10 |
| 82 | 0.01668 | 0.30 | 559.83 | 187.95 | 2.98 | [229800_at](https://www.affymetrix.com/LinkServlet?probeset=229800_at) | [DCLK1](http://www.ncbi.nlm.nih.gov/entrez/query.fcgi?cmd=search&db=gene&term=DCLK1) | doublecortin-like kinase 1 |
| 83 | 0.00590 | 0.25 | 86.71 | 29.11 | 2.98 | [237498_at](https://www.affymetrix.com/LinkServlet?probeset=237498_at) | [NEDD4L](http://www.ncbi.nlm.nih.gov/entrez/query.fcgi?cmd=search&db=gene&term=NEDD4L) | neural precursor cell expressed, developmentally down-regulated 4-like |
| 84 | 0.00636 | 0.26 | 37.32 | 12.53 | 2.98 | [236976_at](https://www.affymetrix.com/LinkServlet?probeset=236976_at) | [FANCA](http://www.ncbi.nlm.nih.gov/entrez/query.fcgi?cmd=search&db=gene&term=FANCA) | Fanconi anemia, complementation group A |
| 85 | 0.01069 | 0.28 | 185.10 | 62.20 | 2.98 | [204974_at](https://www.affymetrix.com/LinkServlet?probeset=204974_at) | [RAB3A](http://www.ncbi.nlm.nih.gov/entrez/query.fcgi?cmd=search&db=gene&term=RAB3A) | RAB3A, member RAS oncogene family |
| 86 | 0.00026 | 0.19 | 821.36 | 276.04 | 2.98 | [204252_at](https://www.affymetrix.com/LinkServlet?probeset=204252_at) | [CDK2](http://www.ncbi.nlm.nih.gov/entrez/query.fcgi?cmd=search&db=gene&term=CDK2) | cyclin-dependent kinase 2 |
| 87 | 0.02164 | 0.32 | 483.02 | 163.62 | 2.95 | [207469_s_at](https://www.affymetrix.com/LinkServlet?probeset=207469_s_at) | [PIR](http://www.ncbi.nlm.nih.gov/entrez/query.fcgi?cmd=search&db=gene&term=PIR) | pirin (iron-binding nuclear protein) |
| 88 | 0.02240 | 0.32 | 90.03 | 30.61 | 2.94 | [215303_at](https://www.affymetrix.com/LinkServlet?probeset=215303_at) | [DCLK1](http://www.ncbi.nlm.nih.gov/entrez/query.fcgi?cmd=search&db=gene&term=DCLK1) | doublecortin-like kinase 1 |
| 89 | 0.04632 | 0.37 | 104.92 | 35.71 | 2.94 | [209590_at](https://www.affymetrix.com/LinkServlet?probeset=209590_at) | [BMP7](http://www.ncbi.nlm.nih.gov/entrez/query.fcgi?cmd=search&db=gene&term=BMP7) | bone morphogenetic protein 7 |
| 90 | 0.00093 | 0.20 | 31.53 | 10.75 | 2.93 | [213816_s_at](https://www.affymetrix.com/LinkServlet?probeset=213816_s_at) | [MET](http://www.ncbi.nlm.nih.gov/entrez/query.fcgi?cmd=search&db=gene&term=MET) | met proto-oncogene (hepatocyte growth factor receptor) |
| 91 | 0.00007 | 0.15 | 19.08 | 6.56 | 2.91 | [210475_at](https://www.affymetrix.com/LinkServlet?probeset=210475_at) | [POU3F1](http://www.ncbi.nlm.nih.gov/entrez/query.fcgi?cmd=search&db=gene&term=POU3F1) | POU class 3 homeobox 1 |
| 92 | 0.02456 | 0.32 | 263.46 | 91.26 | 2.89 | [225531_at](https://www.affymetrix.com/LinkServlet?probeset=225531_at) | [CABLES1](http://www.ncbi.nlm.nih.gov/entrez/query.fcgi?cmd=search&db=gene&term=CABLES1) | Cdk5 and Abl enzyme substrate 1 |
| 93 | 0.02866 | 0.33 | 78.41 | 27.29 | 2.87 | [219042_at](https://www.affymetrix.com/LinkServlet?probeset=219042_at) | [LZTS1](http://www.ncbi.nlm.nih.gov/entrez/query.fcgi?cmd=search&db=gene&term=LZTS1) | leucine zipper, putative tumor suppressor 1 |
| 94 | 0.00040 | 0.19 | 17.39 | 6.07 | 2.86 | [210683_at](https://www.affymetrix.com/LinkServlet?probeset=210683_at) | [NRTN](http://www.ncbi.nlm.nih.gov/entrez/query.fcgi?cmd=search&db=gene&term=NRTN) | neurturin |
| 95 | 0.00390 | 0.24 | 48.41 | 17.01 | 2.85 | [221667_s_at](https://www.affymetrix.com/LinkServlet?probeset=221667_s_at) | [HSPB8](http://www.ncbi.nlm.nih.gov/entrez/query.fcgi?cmd=search&db=gene&term=HSPB8) | heat shock 22kDa protein 8 |
| 96 | 0.02408 | 0.32 | 126.22 | 44.36 | 2.85 | [204044_at](https://www.affymetrix.com/LinkServlet?probeset=204044_at) | [QPRT](http://www.ncbi.nlm.nih.gov/entrez/query.fcgi?cmd=search&db=gene&term=QPRT) | quinolinate phosphoribosyltransferase |
| 97 | 0.00536 | 0.25 | 18.36 | 6.48 | 2.83 | [1553729_s_at](https://www.affymetrix.com/LinkServlet?probeset=1553729_s_at) | [LRRC43](http://www.ncbi.nlm.nih.gov/entrez/query.fcgi?cmd=search&db=gene&term=LRRC43) | leucine rich repeat containing 43 |
| 98 | 0.01915 | 0.31 | 75.91 | 27.02 | 2.81 | [213371_at](https://www.affymetrix.com/LinkServlet?probeset=213371_at) | [LDB3](http://www.ncbi.nlm.nih.gov/entrez/query.fcgi?cmd=search&db=gene&term=LDB3) | LIM domain binding 3 |
| 99 | 0.02147 | 0.32 | 1054.29 | 375.42 | 2.81 | [207233_s_at](https://www.affymetrix.com/LinkServlet?probeset=207233_s_at) | [MITF](http://www.ncbi.nlm.nih.gov/entrez/query.fcgi?cmd=search&db=gene&term=MITF) | microphthalmia-associated transcription factor |
| 100 | 0.01604 | 0.30 | 259.34 | 92.40 | 2.81 | [210964_s_at](https://www.affymetrix.com/LinkServlet?probeset=210964_s_at) | [GYG2](http://www.ncbi.nlm.nih.gov/entrez/query.fcgi?cmd=search&db=gene&term=GYG2) | glycogenin 2 |
| 101 | 0.03498 | 0.35 | 3926.09 | 1405.03 | 2.79 | [204653_at](https://www.affymetrix.com/LinkServlet?probeset=204653_at) | [TFAP2A](http://www.ncbi.nlm.nih.gov/entrez/query.fcgi?cmd=search&db=gene&term=TFAP2A) | transcription factor AP-2 alpha (activating enhancer binding protein 2 alpha) |
| 102 | 0.00656 | 0.26 | 36.74 | 13.15 | 2.79 | [232421_at](https://www.affymetrix.com/LinkServlet?probeset=232421_at) | [SCARB1](http://www.ncbi.nlm.nih.gov/entrez/query.fcgi?cmd=search&db=gene&term=SCARB1) | scavenger receptor class B, member 1 |
| 103 | 0.01264 | 0.29 | 316.84 | 113.54 | 2.79 | [205862_at](https://www.affymetrix.com/LinkServlet?probeset=205862_at) | [GREB1](http://www.ncbi.nlm.nih.gov/entrez/query.fcgi?cmd=search&db=gene&term=GREB1) | GREB1 protein |
| 104 | 0.00017 | 0.18 | 23.22 | 8.35 | 2.78 | [217124_at](https://www.affymetrix.com/LinkServlet?probeset=217124_at) | [IQCE](http://www.ncbi.nlm.nih.gov/entrez/query.fcgi?cmd=search&db=gene&term=IQCE) | IQ motif containing E |
| 105 | 0.00793 | 0.27 | 86.84 | 31.22 | 2.78 | [206114_at](https://www.affymetrix.com/LinkServlet?probeset=206114_at) | [EPHA4](http://www.ncbi.nlm.nih.gov/entrez/query.fcgi?cmd=search&db=gene&term=EPHA4) | EPH receptor A4 |
| 106 | 0.00444 | 0.24 | 194.08 | 70.37 | 2.76 | [213924_at](https://www.affymetrix.com/LinkServlet?probeset=213924_at) | [MPPE1](http://www.ncbi.nlm.nih.gov/entrez/query.fcgi?cmd=search&db=gene&term=MPPE1) | metallophosphoesterase 1 |
| 107 | 0.00494 | 0.25 | 476.40 | 172.77 | 2.76 | [212445_s_at](https://www.affymetrix.com/LinkServlet?probeset=212445_s_at) | [NEDD4L](http://www.ncbi.nlm.nih.gov/entrez/query.fcgi?cmd=search&db=gene&term=NEDD4L) | neural precursor cell expressed, developmentally down-regulated 4-like |
| 108 | 0.03581 | 0.35 | 90.34 | 32.76 | 2.76 | [232282_at](https://www.affymetrix.com/LinkServlet?probeset=232282_at) | [WNK3](http://www.ncbi.nlm.nih.gov/entrez/query.fcgi?cmd=search&db=gene&term=WNK3) | WNK lysine deficient protein kinase 3 |
| 109 | 0.01925 | 0.31 | 108.59 | 39.48 | 2.75 | [63305_at](https://www.affymetrix.com/LinkServlet?probeset=63305_at) | [PKNOX2](http://www.ncbi.nlm.nih.gov/entrez/query.fcgi?cmd=search&db=gene&term=PKNOX2) | PBX/knotted 1 homeobox 2 |
| 110 | 0.00609 | 0.26 | 89.69 | 32.68 | 2.74 | [235572_at](https://www.affymetrix.com/LinkServlet?probeset=235572_at) | [SPC24](http://www.ncbi.nlm.nih.gov/entrez/query.fcgi?cmd=search&db=gene&term=SPC24) | SPC24, NDC80 kinetochore complex component, homolog (S. cerevisiae) |
| 111 | 0.00168 | 0.20 | 195.22 | 71.37 | 2.74 | [218631_at](https://www.affymetrix.com/LinkServlet?probeset=218631_at) | [AVPI1](http://www.ncbi.nlm.nih.gov/entrez/query.fcgi?cmd=search&db=gene&term=AVPI1) | arginine vasopressin-induced 1 |
| 112 | 0.04445 | 0.37 | 140.42 | 51.39 | 2.73 | [209843_s_at](https://www.affymetrix.com/LinkServlet?probeset=209843_s_at) | [SOX10](http://www.ncbi.nlm.nih.gov/entrez/query.fcgi?cmd=search&db=gene&term=SOX10) | SRY (sex determining region Y)-box 10 |
| 113 | 0.02034 | 0.31 | 894.92 | 328.15 | 2.73 | [222760_at](https://www.affymetrix.com/LinkServlet?probeset=222760_at) | [ZNF703](http://www.ncbi.nlm.nih.gov/entrez/query.fcgi?cmd=search&db=gene&term=ZNF703) | zinc finger protein 703 |
| 114 | 0.02211 | 0.32 | 533.01 | 195.57 | 2.73 | [210613_s_at](https://www.affymetrix.com/LinkServlet?probeset=210613_s_at) | [SYNGR1](http://www.ncbi.nlm.nih.gov/entrez/query.fcgi?cmd=search&db=gene&term=SYNGR1) | synaptogyrin 1 |
| 115 | 0.02851 | 0.33 | 225.07 | 82.84 | 2.72 | [230972_at](https://www.affymetrix.com/LinkServlet?probeset=230972_at) | [ANKRD9](http://www.ncbi.nlm.nih.gov/entrez/query.fcgi?cmd=search&db=gene&term=ANKRD9) | ankyrin repeat domain 9 |
| 116 | 0.02405 | 0.32 | 39.30 | 14.49 | 2.71 | [231558_at](https://www.affymetrix.com/LinkServlet?probeset=231558_at) | [INSM1](http://www.ncbi.nlm.nih.gov/entrez/query.fcgi?cmd=search&db=gene&term=INSM1) | insulinoma-associated 1 |
| 117 | 0.00068 | 0.20 | 16.34 | 6.03 | 2.71 | [235465_at](https://www.affymetrix.com/LinkServlet?probeset=235465_at) | [FAM123A](http://www.ncbi.nlm.nih.gov/entrez/query.fcgi?cmd=search&db=gene&term=FAM123A) | family with sequence similarity 123A |
| 118 | 0.00156 | 0.20 | 93.76 | 34.72 | 2.70 | [203126_at](https://www.affymetrix.com/LinkServlet?probeset=203126_at) | [IMPA2](http://www.ncbi.nlm.nih.gov/entrez/query.fcgi?cmd=search&db=gene&term=IMPA2) | inositol(myo)-1(or 4)-monophosphatase 2 |
| 119 | 0.04899 | 0.38 | 172.64 | 64.04 | 2.70 | [213478_at](https://www.affymetrix.com/LinkServlet?probeset=213478_at) | [RP1-21O18.1](http://www.ncbi.nlm.nih.gov/entrez/query.fcgi?cmd=search&db=gene&term=RP1-21O18.1) | kazrin |
| 120 | 0.00049 | 0.19 | 26.52 | 9.85 | 2.69 | [211804_s_at](https://www.affymetrix.com/LinkServlet?probeset=211804_s_at) | [CDK2](http://www.ncbi.nlm.nih.gov/entrez/query.fcgi?cmd=search&db=gene&term=CDK2) | cyclin-dependent kinase 2 |
| 121 | 0.00667 | 0.26 | 16.83 | 6.25 | 2.69 | [1558846_at](https://www.affymetrix.com/LinkServlet?probeset=1558846_at) | [PNLIPRP3](http://www.ncbi.nlm.nih.gov/entrez/query.fcgi?cmd=search&db=gene&term=PNLIPRP3) | pancreatic lipase-related protein 3 |
| 122 | 0.01649 | 0.30 | 185.99 | 69.21 | 2.69 | [229003_x_at](https://www.affymetrix.com/LinkServlet?probeset=229003_x_at) | [FAM69B](http://www.ncbi.nlm.nih.gov/entrez/query.fcgi?cmd=search&db=gene&term=FAM69B) | family with sequence similarity 69, member B |
| 123 | 0.00803 | 0.27 | 93.44 | 34.91 | 2.68 | [225025_at](https://www.affymetrix.com/LinkServlet?probeset=225025_at) | [IGSF8](http://www.ncbi.nlm.nih.gov/entrez/query.fcgi?cmd=search&db=gene&term=IGSF8) | immunoglobulin superfamily, member 8 |
| 124 | 0.01610 | 0.30 | 150.04 | 56.20 | 2.67 | [204562_at](https://www.affymetrix.com/LinkServlet?probeset=204562_at) | [IRF4](http://www.ncbi.nlm.nih.gov/entrez/query.fcgi?cmd=search&db=gene&term=IRF4) | interferon regulatory factor 4 |
| 125 | 0.03972 | 0.36 | 110.05 | 41.35 | 2.66 | [210854_x_at](https://www.affymetrix.com/LinkServlet?probeset=210854_x_at) | [SLC6A8](http://www.ncbi.nlm.nih.gov/entrez/query.fcgi?cmd=search&db=gene&term=SLC6A8) | solute carrier family 6 (neurotransmitter transporter, creatine), member 8 |
| 126 | 0.00583 | 0.25 | 87.92 | 33.06 | 2.66 | [229251_s_at](https://www.affymetrix.com/LinkServlet?probeset=229251_s_at) | [TPCN2](http://www.ncbi.nlm.nih.gov/entrez/query.fcgi?cmd=search&db=gene&term=TPCN2) | two pore segment channel 2 |
| 127 | 0.01490 | 0.29 | 54.63 | 20.54 | 2.66 | [244623_at](https://www.affymetrix.com/LinkServlet?probeset=244623_at) | [KCNQ5](http://www.ncbi.nlm.nih.gov/entrez/query.fcgi?cmd=search&db=gene&term=KCNQ5) | potassium voltage-gated channel, KQT-like subfamily, member 5 |
| 128 | 0.01730 | 0.30 | 143.76 | 54.08 | 2.66 | [222171_s_at](https://www.affymetrix.com/LinkServlet?probeset=222171_s_at) | [PKNOX2](http://www.ncbi.nlm.nih.gov/entrez/query.fcgi?cmd=search&db=gene&term=PKNOX2) | PBX/knotted 1 homeobox 2 |
| 129 | 0.00436 | 0.24 | 144.55 | 54.39 | 2.66 | [229250_at](https://www.affymetrix.com/LinkServlet?probeset=229250_at) | [TPCN2](http://www.ncbi.nlm.nih.gov/entrez/query.fcgi?cmd=search&db=gene&term=TPCN2) | two pore segment channel 2 |
| 130 | 0.01060 | 0.28 | 46.81 | 17.62 | 2.66 | [205142_x_at](https://www.affymetrix.com/LinkServlet?probeset=205142_x_at) | [ABCD1](http://www.ncbi.nlm.nih.gov/entrez/query.fcgi?cmd=search&db=gene&term=ABCD1) | ATP-binding cassette, sub-family D (ALD), member 1 |
| 131 | 0.02937 | 0.34 | 113.09 | 42.57 | 2.66 | [213843_x_at](https://www.affymetrix.com/LinkServlet?probeset=213843_x_at) | [SLC6A8](http://www.ncbi.nlm.nih.gov/entrez/query.fcgi?cmd=search&db=gene&term=SLC6A8) | solute carrier family 6 (neurotransmitter transporter, creatine), member 8 |
| 132 | 0.00987 | 0.28 | 30.41 | 11.49 | 2.65 | [233057_at](https://www.affymetrix.com/LinkServlet?probeset=233057_at) | [HSPB8](http://www.ncbi.nlm.nih.gov/entrez/query.fcgi?cmd=search&db=gene&term=HSPB8) | heat shock 22kDa protein 8 |
| 133 | 0.00354 | 0.24 | 443.45 | 167.50 | 2.65 | [202580_x_at](https://www.affymetrix.com/LinkServlet?probeset=202580_x_at) | [FOXM1](http://www.ncbi.nlm.nih.gov/entrez/query.fcgi?cmd=search&db=gene&term=FOXM1) | forkhead box M1 |
| 134 | 0.02126 | 0.32 | 125.13 | 47.50 | 2.63 | [215812_s_at](https://www.affymetrix.com/LinkServlet?probeset=215812_s_at) | [SLC6A10P](http://www.ncbi.nlm.nih.gov/entrez/query.fcgi?cmd=search&db=gene&term=SLC6A10P) | solute carrier family 6 (neurotransmitter transporter, creatine), member 10 (pseudogene) |
| 135 | 0.02354 | 0.32 | 20.43 | 7.77 | 2.63 | [207789_s_at](https://www.affymetrix.com/LinkServlet?probeset=207789_s_at) | [DPP6](http://www.ncbi.nlm.nih.gov/entrez/query.fcgi?cmd=search&db=gene&term=DPP6) | dipeptidyl-peptidase 6 |
| 136 | 0.03225 | 0.34 | 79.39 | 30.30 | 2.62 | [211549_s_at](https://www.affymetrix.com/LinkServlet?probeset=211549_s_at) | [HPGD](http://www.ncbi.nlm.nih.gov/entrez/query.fcgi?cmd=search&db=gene&term=HPGD) | hydroxyprostaglandin dehydrogenase 15-(NAD) |
| 137 | 0.00040 | 0.19 | 36.72 | 14.09 | 2.61 | [216979_at](https://www.affymetrix.com/LinkServlet?probeset=216979_at) | [NR4A3](http://www.ncbi.nlm.nih.gov/entrez/query.fcgi?cmd=search&db=gene&term=NR4A3) | nuclear receptor subfamily 4, group A, member 3 |
| 138 | 0.02545 | 0.33 | 545.07 | 209.23 | 2.61 | [229002_at](https://www.affymetrix.com/LinkServlet?probeset=229002_at) | [FAM69B](http://www.ncbi.nlm.nih.gov/entrez/query.fcgi?cmd=search&db=gene&term=FAM69B) | family with sequence similarity 69, member B |
| 139 | 0.00150 | 0.20 | 45.97 | 17.71 | 2.60 | [1563839_at](https://www.affymetrix.com/LinkServlet?probeset=1563839_at) | [TBC1D7](http://www.ncbi.nlm.nih.gov/entrez/query.fcgi?cmd=search&db=gene&term=TBC1D7) | TBC1 domain family, member 7 |
| 140 | 0.01719 | 0.30 | 121.66 | 47.05 | 2.59 | [235334_at](https://www.affymetrix.com/LinkServlet?probeset=235334_at) | [ST6GALNAC3](http://www.ncbi.nlm.nih.gov/entrez/query.fcgi?cmd=search&db=gene&term=ST6GALNAC3) | ST6 (alpha-N-acetyl-neuraminyl-2,3-beta-galactosyl-1,3)-N-acetylgalactosaminide alpha-2,6-sialyltransferase 3 |
| 141 | 0.00217 | 0.22 | 45.17 | 17.55 | 2.57 | [206972_s_at](https://www.affymetrix.com/LinkServlet?probeset=206972_s_at) | [GPR161](http://www.ncbi.nlm.nih.gov/entrez/query.fcgi?cmd=search&db=gene&term=GPR161) | G protein-coupled receptor 161 |
| 142 | 0.00201 | 0.21 | 48.80 | 18.96 | 2.57 | [230145_at](https://www.affymetrix.com/LinkServlet?probeset=230145_at) | [DUS3L](http://www.ncbi.nlm.nih.gov/entrez/query.fcgi?cmd=search&db=gene&term=DUS3L) | dihydrouridine synthase 3-like (S. cerevisiae) |
| 143 | 0.04716 | 0.38 | 1003.93 | 391.25 | 2.57 | [212070_at](https://www.affymetrix.com/LinkServlet?probeset=212070_at) | [GPR56](http://www.ncbi.nlm.nih.gov/entrez/query.fcgi?cmd=search&db=gene&term=GPR56) | G protein-coupled receptor 56 |
| 144 | 0.01341 | 0.29 | 22.14 | 8.67 | 2.55 | [230496_at](https://www.affymetrix.com/LinkServlet?probeset=230496_at) | [FAM123A](http://www.ncbi.nlm.nih.gov/entrez/query.fcgi?cmd=search&db=gene&term=FAM123A) | family with sequence similarity 123A |
| 145 | 0.03477 | 0.35 | 117.53 | 46.22 | 2.54 | [1554701_a_at](https://www.affymetrix.com/LinkServlet?probeset=1554701_a_at) | [TBC1D16](http://www.ncbi.nlm.nih.gov/entrez/query.fcgi?cmd=search&db=gene&term=TBC1D16) | TBC1 domain family, member 16 |
| 146 | 0.00018 | 0.18 | 25.94 | 10.20 | 2.54 | [215834_x_at](https://www.affymetrix.com/LinkServlet?probeset=215834_x_at) | [SCARB1](http://www.ncbi.nlm.nih.gov/entrez/query.fcgi?cmd=search&db=gene&term=SCARB1) | scavenger receptor class B, member 1 |
| 147 | 0.01954 | 0.31 | 558.37 | 219.84 | 2.54 | [224735_at](https://www.affymetrix.com/LinkServlet?probeset=224735_at) | [CYBASC3](http://www.ncbi.nlm.nih.gov/entrez/query.fcgi?cmd=search&db=gene&term=CYBASC3) | cytochrome b, ascorbate dependent 3 |
| 148 | 0.03051 | 0.34 | 27.74 | 10.93 | 2.54 | [229925_at](https://www.affymetrix.com/LinkServlet?probeset=229925_at) | [SLC6A17](http://www.ncbi.nlm.nih.gov/entrez/query.fcgi?cmd=search&db=gene&term=SLC6A17) | solute carrier family 6, member 17 |
| 149 | 0.00126 | 0.20 | 35.51 | 14.03 | 2.53 | [201710_at](https://www.affymetrix.com/LinkServlet?probeset=201710_at) | [MYBL2](http://www.ncbi.nlm.nih.gov/entrez/query.fcgi?cmd=search&db=gene&term=MYBL2) | v-myb myeloblastosis viral oncogene homolog (avian)-like 2 |
| 150 | 0.00770 | 0.27 | 80.15 | 31.72 | 2.53 | [211752_s_at](https://www.affymetrix.com/LinkServlet?probeset=211752_s_at) | [NDUFS7](http://www.ncbi.nlm.nih.gov/entrez/query.fcgi?cmd=search&db=gene&term=NDUFS7) | NADH dehydrogenase (ubiquinone) Fe-S protein 7, 20kDa (NADH-coenzyme Q reductase) |
| 151 | 0.00304 | 0.23 | 20.91 | 8.29 | 2.52 | [228550_at](https://www.affymetrix.com/LinkServlet?probeset=228550_at) | [RTN4R](http://www.ncbi.nlm.nih.gov/entrez/query.fcgi?cmd=search&db=gene&term=RTN4R) | reticulon 4 receptor |
| 152 | 0.01122 | 0.28 | 73.81 | 29.35 | 2.51 | [221909_at](https://www.affymetrix.com/LinkServlet?probeset=221909_at) | [RNFT2](http://www.ncbi.nlm.nih.gov/entrez/query.fcgi?cmd=search&db=gene&term=RNFT2) | ring finger protein, transmembrane 2 |
| 153 | 0.02741 | 0.33 | 23.74 | 9.45 | 2.51 | [243209_at](https://www.affymetrix.com/LinkServlet?probeset=243209_at) | [KCNQ4](http://www.ncbi.nlm.nih.gov/entrez/query.fcgi?cmd=search&db=gene&term=KCNQ4) | potassium voltage-gated channel, KQT-like subfamily, member 4 |
| 154 | 0.00110 | 0.20 | 56.08 | 22.34 | 2.51 | [224461_s_at](https://www.affymetrix.com/LinkServlet?probeset=224461_s_at) | [AIFM2](http://www.ncbi.nlm.nih.gov/entrez/query.fcgi?cmd=search&db=gene&term=AIFM2) | apoptosis-inducing factor, mitochondrion-associated, 2 |
| 155 | 0.01171 | 0.28 | 53.19 | 133.32 | 0.40 | [204285_s_at](https://www.affymetrix.com/LinkServlet?probeset=204285_s_at) | [PMAIP1](http://www.ncbi.nlm.nih.gov/entrez/query.fcgi?cmd=search&db=gene&term=PMAIP1) | phorbol-12-myristate-13-acetate-induced protein 1 |
| 156 | 0.00698 | 0.26 | 93.64 | 235.19 | 0.40 | [222033_s_at](https://www.affymetrix.com/LinkServlet?probeset=222033_s_at) | [FLT1](http://www.ncbi.nlm.nih.gov/entrez/query.fcgi?cmd=search&db=gene&term=FLT1) | fms-related tyrosine kinase 1 (vascular endothelial growth factor/vascular permeability factor receptor) |
| 157 | 0.01162 | 0.28 | 128.48 | 322.77 | 0.40 | [203787_at](https://www.affymetrix.com/LinkServlet?probeset=203787_at) | [SSBP2](http://www.ncbi.nlm.nih.gov/entrez/query.fcgi?cmd=search&db=gene&term=SSBP2) | single-stranded DNA binding protein 2 |
| 158 | 0.03904 | 0.36 | 274.12 | 690.93 | 0.40 | [201832_s_at](https://www.affymetrix.com/LinkServlet?probeset=201832_s_at) | [USO1](http://www.ncbi.nlm.nih.gov/entrez/query.fcgi?cmd=search&db=gene&term=USO1) | USO1 homolog, vesicle docking protein (yeast) |
| 159 | 0.00933 | 0.27 | 191.12 | 481.94 | 0.40 | [223220_s_at](https://www.affymetrix.com/LinkServlet?probeset=223220_s_at) | [PARP9](http://www.ncbi.nlm.nih.gov/entrez/query.fcgi?cmd=search&db=gene&term=PARP9) | poly (ADP-ribose) polymerase family, member 9 |
| 160 | 0.00746 | 0.27 | 617.42 | 1559.13 | 0.40 | [202133_at](https://www.affymetrix.com/LinkServlet?probeset=202133_at) | [WWTR1](http://www.ncbi.nlm.nih.gov/entrez/query.fcgi?cmd=search&db=gene&term=WWTR1) | WW domain containing transcription regulator 1 |
| 161 | 0.01822 | 0.31 | 21.96 | 55.58 | 0.40 | [216233_at](https://www.affymetrix.com/LinkServlet?probeset=216233_at) | [CD163](http://www.ncbi.nlm.nih.gov/entrez/query.fcgi?cmd=search&db=gene&term=CD163) | CD163 molecule |
| 162 | 0.01677 | 0.30 | 213.57 | 541.64 | 0.39 | [213850_s_at](https://www.affymetrix.com/LinkServlet?probeset=213850_s_at) | [SFRS2IP](http://www.ncbi.nlm.nih.gov/entrez/query.fcgi?cmd=search&db=gene&term=SFRS2IP) | splicing factor, arginine/serine-rich 2, interacting protein |
| 163 | 0.01341 | 0.29 | 189.44 | 482.43 | 0.39 | [201581_at](https://www.affymetrix.com/LinkServlet?probeset=201581_at) | [TXNDC13](http://www.ncbi.nlm.nih.gov/entrez/query.fcgi?cmd=search&db=gene&term=TXNDC13) | thioredoxin domain containing 13 |
| 164 | 0.02005 | 0.31 | 379.41 | 968.11 | 0.39 | [209684_at](https://www.affymetrix.com/LinkServlet?probeset=209684_at) | [RIN2](http://www.ncbi.nlm.nih.gov/entrez/query.fcgi?cmd=search&db=gene&term=RIN2) | Ras and Rab interactor 2 |
| 165 | 0.03065 | 0.34 | 76.27 | 194.96 | 0.39 | [235306_at](https://www.affymetrix.com/LinkServlet?probeset=235306_at) | [GIMAP8](http://www.ncbi.nlm.nih.gov/entrez/query.fcgi?cmd=search&db=gene&term=GIMAP8) | GTPase, IMAP family member 8 |
| 166 | 0.03037 | 0.34 | 23.73 | 60.76 | 0.39 | [223313_s_at](https://www.affymetrix.com/LinkServlet?probeset=223313_s_at) | [MAGED4B](http://www.ncbi.nlm.nih.gov/entrez/query.fcgi?cmd=search&db=gene&term=MAGED4B) | melanoma antigen family D, 4B |
| 167 | 0.00403 | 0.24 | 320.94 | 823.40 | 0.39 | [201645_at](https://www.affymetrix.com/LinkServlet?probeset=201645_at) | [TNC](http://www.ncbi.nlm.nih.gov/entrez/query.fcgi?cmd=search&db=gene&term=TNC) | tenascin C |
| 168 | 0.00522 | 0.25 | 76.71 | 197.66 | 0.39 | [213839_at](https://www.affymetrix.com/LinkServlet?probeset=213839_at) | [CLMN](http://www.ncbi.nlm.nih.gov/entrez/query.fcgi?cmd=search&db=gene&term=CLMN) | calmin (calponin-like, transmembrane) |
| 169 | 0.00392 | 0.24 | 36.76 | 94.84 | 0.39 | [220301_at](https://www.affymetrix.com/LinkServlet?probeset=220301_at) | [CCDC102B](http://www.ncbi.nlm.nih.gov/entrez/query.fcgi?cmd=search&db=gene&term=CCDC102B) | coiled-coil domain containing 102B |
| 170 | 0.03037 | 0.34 | 947.42 | 2445.13 | 0.39 | [200599_s_at](https://www.affymetrix.com/LinkServlet?probeset=200599_s_at) | [HSP90B1](http://www.ncbi.nlm.nih.gov/entrez/query.fcgi?cmd=search&db=gene&term=HSP90B1) | heat shock protein 90kDa beta (Grp94), member 1 |
| 171 | 0.01773 | 0.30 | 15.98 | 41.26 | 0.39 | [206091_at](https://www.affymetrix.com/LinkServlet?probeset=206091_at) | [MATN3](http://www.ncbi.nlm.nih.gov/entrez/query.fcgi?cmd=search&db=gene&term=MATN3) | matrilin 3 |
| 172 | 0.00927 | 0.27 | 37.07 | 96.10 | 0.39 | [204924_at](https://www.affymetrix.com/LinkServlet?probeset=204924_at) | [TLR2](http://www.ncbi.nlm.nih.gov/entrez/query.fcgi?cmd=search&db=gene&term=TLR2) | toll-like receptor 2 |
| 173 | 0.01992 | 0.31 | 196.55 | 511.25 | 0.38 | [204872_at](https://www.affymetrix.com/LinkServlet?probeset=204872_at) | [TLE4](http://www.ncbi.nlm.nih.gov/entrez/query.fcgi?cmd=search&db=gene&term=TLE4) | transducin-like enhancer of split 4 (E(sp1) homolog, Drosophila) |
| 174 | 0.04655 | 0.38 | 25.10 | 65.33 | 0.38 | [222780_s_at](https://www.affymetrix.com/LinkServlet?probeset=222780_s_at) | [BAALC](http://www.ncbi.nlm.nih.gov/entrez/query.fcgi?cmd=search&db=gene&term=BAALC) | brain and acute leukemia, cytoplasmic |
| 175 | 0.02444 | 0.32 | 28.34 | 73.78 | 0.38 | [204749_at](https://www.affymetrix.com/LinkServlet?probeset=204749_at) | [NAP1L3](http://www.ncbi.nlm.nih.gov/entrez/query.fcgi?cmd=search&db=gene&term=NAP1L3) | nucleosome assembly protein 1-like 3 |
| 176 | 0.04753 | 0.38 | 12.43 | 32.38 | 0.38 | [218720_x_at](https://www.affymetrix.com/LinkServlet?probeset=218720_x_at) | [SEZ6L2](http://www.ncbi.nlm.nih.gov/entrez/query.fcgi?cmd=search&db=gene&term=SEZ6L2) | seizure related 6 homolog (mouse)-like 2 |
| 177 | 0.00542 | 0.25 | 23.94 | 62.42 | 0.38 | [224218_s_at](https://www.affymetrix.com/LinkServlet?probeset=224218_s_at) | [TRPS1](http://www.ncbi.nlm.nih.gov/entrez/query.fcgi?cmd=search&db=gene&term=TRPS1) | trichorhinophalangeal syndrome I |
| 178 | 0.04330 | 0.37 | 296.74 | 774.68 | 0.38 | [224736_at](https://www.affymetrix.com/LinkServlet?probeset=224736_at) | [CCAR1](http://www.ncbi.nlm.nih.gov/entrez/query.fcgi?cmd=search&db=gene&term=CCAR1) | cell division cycle and apoptosis regulator 1 |
| 179 | 0.02703 | 0.33 | 497.03 | 1307.38 | 0.38 | [232382_s_at](https://www.affymetrix.com/LinkServlet?probeset=232382_s_at) | [PCMTD1](http://www.ncbi.nlm.nih.gov/entrez/query.fcgi?cmd=search&db=gene&term=PCMTD1) | protein-L-isoaspartate (D-aspartate) O-methyltransferase domain containing 1 |
| 180 | 0.03960 | 0.36 | 488.20 | 1285.47 | 0.38 | [202061_s_at](https://www.affymetrix.com/LinkServlet?probeset=202061_s_at) | [SEL1L](http://www.ncbi.nlm.nih.gov/entrez/query.fcgi?cmd=search&db=gene&term=SEL1L) | sel-1 suppressor of lin-12-like (C. elegans) |
| 181 | 0.02874 | 0.33 | 384.37 | 1014.66 | 0.38 | [218649_x_at](https://www.affymetrix.com/LinkServlet?probeset=218649_x_at) | [SDCCAG1](http://www.ncbi.nlm.nih.gov/entrez/query.fcgi?cmd=search&db=gene&term=SDCCAG1) | serologically defined colon cancer antigen 1 |
| 182 | 0.04698 | 0.38 | 186.62 | 493.86 | 0.38 | [211651_s_at](https://www.affymetrix.com/LinkServlet?probeset=211651_s_at) | [LAMB1](http://www.ncbi.nlm.nih.gov/entrez/query.fcgi?cmd=search&db=gene&term=LAMB1) | laminin, beta 1 |
| 183 | 0.04926 | 0.38 | 11.23 | 29.85 | 0.38 | [202035_s_at](https://www.affymetrix.com/LinkServlet?probeset=202035_s_at) | [SFRP1](http://www.ncbi.nlm.nih.gov/entrez/query.fcgi?cmd=search&db=gene&term=SFRP1) | secreted frizzled-related protein 1 |
| 184 | 0.00549 | 0.25 | 25.59 | 68.20 | 0.38 | [221211_s_at](https://www.affymetrix.com/LinkServlet?probeset=221211_s_at) | [C21orf7](http://www.ncbi.nlm.nih.gov/entrez/query.fcgi?cmd=search&db=gene&term=C21orf7) | chromosome 21 open reading frame 7 |
| 185 | 0.02129 | 0.32 | 285.54 | 763.42 | 0.37 | [201604_s_at](https://www.affymetrix.com/LinkServlet?probeset=201604_s_at) | [PPP1R12A](http://www.ncbi.nlm.nih.gov/entrez/query.fcgi?cmd=search&db=gene&term=PPP1R12A) | protein phosphatase 1, regulatory (inhibitor) subunit 12A |
| 186 | 0.03699 | 0.36 | 36.33 | 97.19 | 0.37 | [214469_at](https://www.affymetrix.com/LinkServlet?probeset=214469_at) | [HIST1H2AE](http://www.ncbi.nlm.nih.gov/entrez/query.fcgi?cmd=search&db=gene&term=HIST1H2AE) | histone cluster 1, H2ae |
| 187 | 0.00144 | 0.20 | 238.30 | 637.56 | 0.37 | [223092_at](https://www.affymetrix.com/LinkServlet?probeset=223092_at) | [ANKH](http://www.ncbi.nlm.nih.gov/entrez/query.fcgi?cmd=search&db=gene&term=ANKH) | ankylosis, progressive homolog (mouse) |
| 188 | 0.02366 | 0.32 | 137.43 | 367.92 | 0.37 | [225612_s_at](https://www.affymetrix.com/LinkServlet?probeset=225612_s_at) | [B3GNT5](http://www.ncbi.nlm.nih.gov/entrez/query.fcgi?cmd=search&db=gene&term=B3GNT5) | UDP-GlcNAc:betaGal beta-1,3-N-acetylglucosaminyltransferase 5 |
| 189 | 0.03490 | 0.35 | 256.92 | 688.81 | 0.37 | [213293_s_at](https://www.affymetrix.com/LinkServlet?probeset=213293_s_at) | [TRIM22](http://www.ncbi.nlm.nih.gov/entrez/query.fcgi?cmd=search&db=gene&term=TRIM22) | tripartite motif-containing 22 |
| 190 | 0.02845 | 0.33 | 169.07 | 453.87 | 0.37 | [226757_at](https://www.affymetrix.com/LinkServlet?probeset=226757_at) | [IFIT2](http://www.ncbi.nlm.nih.gov/entrez/query.fcgi?cmd=search&db=gene&term=IFIT2) | interferon-induced protein with tetratricopeptide repeats 2 |
| 191 | 0.04281 | 0.37 | 169.31 | 454.86 | 0.37 | [204472_at](https://www.affymetrix.com/LinkServlet?probeset=204472_at) | [GEM](http://www.ncbi.nlm.nih.gov/entrez/query.fcgi?cmd=search&db=gene&term=GEM) | GTP binding protein overexpressed in skeletal muscle |
| 192 | 0.02456 | 0.32 | 292.56 | 786.05 | 0.37 | [225202_at](https://www.affymetrix.com/LinkServlet?probeset=225202_at) | [RHOBTB3](http://www.ncbi.nlm.nih.gov/entrez/query.fcgi?cmd=search&db=gene&term=RHOBTB3) | Rho-related BTB domain containing 3 |
| 193 | 0.02632 | 0.33 | 294.21 | 791.05 | 0.37 | [204438_at](https://www.affymetrix.com/LinkServlet?probeset=204438_at) | [MRC1](http://www.ncbi.nlm.nih.gov/entrez/query.fcgi?cmd=search&db=gene&term=MRC1) | mannose receptor, C type 1 |
| 194 | 0.01097 | 0.28 | 549.25 | 1478.79 | 0.37 | [225093_at](https://www.affymetrix.com/LinkServlet?probeset=225093_at) | [UTRN](http://www.ncbi.nlm.nih.gov/entrez/query.fcgi?cmd=search&db=gene&term=UTRN) | utrophin |
| 195 | 0.00164 | 0.20 | 43.90 | 118.20 | 0.37 | [223533_at](https://www.affymetrix.com/LinkServlet?probeset=223533_at) | [LRRC8C](http://www.ncbi.nlm.nih.gov/entrez/query.fcgi?cmd=search&db=gene&term=LRRC8C) | leucine rich repeat containing 8 family, member C |
| 196 | 0.02631 | 0.33 | 13.89 | 37.49 | 0.37 | [228608_at](https://www.affymetrix.com/LinkServlet?probeset=228608_at) | [NALCN](http://www.ncbi.nlm.nih.gov/entrez/query.fcgi?cmd=search&db=gene&term=NALCN) | sodium leak channel, non-selective |
| 197 | 0.01893 | 0.31 | 10.61 | 28.67 | 0.37 | [1552575_a_at](https://www.affymetrix.com/LinkServlet?probeset=1552575_a_at) | [C6orf141](http://www.ncbi.nlm.nih.gov/entrez/query.fcgi?cmd=search&db=gene&term=C6orf141) | chromosome 6 open reading frame 141 |
| 198 | 0.01244 | 0.29 | 182.52 | 493.13 | 0.37 | [205003_at](https://www.affymetrix.com/LinkServlet?probeset=205003_at) | [DOCK4](http://www.ncbi.nlm.nih.gov/entrez/query.fcgi?cmd=search&db=gene&term=DOCK4) | dedicator of cytokinesis 4 |
| 199 | 0.02420 | 0.32 | 11.79 | 31.93 | 0.37 | [243231_at](https://www.affymetrix.com/LinkServlet?probeset=243231_at) | [SLC38A11](http://www.ncbi.nlm.nih.gov/entrez/query.fcgi?cmd=search&db=gene&term=SLC38A11) | solute carrier family 38, member 11 |
| 200 | 0.01498 | 0.29 | 131.07 | 355.07 | 0.37 | [218729_at](https://www.affymetrix.com/LinkServlet?probeset=218729_at) | [LXN](http://www.ncbi.nlm.nih.gov/entrez/query.fcgi?cmd=search&db=gene&term=LXN) | latexin |
| 201 | 0.01482 | 0.29 | 269.93 | 732.99 | 0.37 | [214247_s_at](https://www.affymetrix.com/LinkServlet?probeset=214247_s_at) | [DKK3](http://www.ncbi.nlm.nih.gov/entrez/query.fcgi?cmd=search&db=gene&term=DKK3) | dickkopf homolog 3 (Xenopus laevis) |
| 202 | 0.01046 | 0.28 | 32.38 | 87.98 | 0.37 | [221185_s_at](https://www.affymetrix.com/LinkServlet?probeset=221185_s_at) | [IQCG](http://www.ncbi.nlm.nih.gov/entrez/query.fcgi?cmd=search&db=gene&term=IQCG) | IQ motif containing G |
| 203 | 0.04145 | 0.37 | 262.44 | 713.18 | 0.37 | [225864_at](https://www.affymetrix.com/LinkServlet?probeset=225864_at) | [FAM84B](http://www.ncbi.nlm.nih.gov/entrez/query.fcgi?cmd=search&db=gene&term=FAM84B) | family with sequence similarity 84, member B |
| 204 | 0.04208 | 0.37 | 40.62 | 110.60 | 0.37 | [218899_s_at](https://www.affymetrix.com/LinkServlet?probeset=218899_s_at) | [BAALC](http://www.ncbi.nlm.nih.gov/entrez/query.fcgi?cmd=search&db=gene&term=BAALC) | brain and acute leukemia, cytoplasmic |
| 205 | 0.04256 | 0.37 | 53.45 | 145.97 | 0.37 | [222731_at](https://www.affymetrix.com/LinkServlet?probeset=222731_at) | [ZDHHC2](http://www.ncbi.nlm.nih.gov/entrez/query.fcgi?cmd=search&db=gene&term=ZDHHC2) | zinc finger, DHHC-type containing 2 |
| 206 | 0.03884 | 0.36 | 6.59 | 18.01 | 0.37 | [242344_at](https://www.affymetrix.com/LinkServlet?probeset=242344_at) | [GABRB2](http://www.ncbi.nlm.nih.gov/entrez/query.fcgi?cmd=search&db=gene&term=GABRB2) | gamma-aminobutyric acid (GABA) A receptor, beta 2 |
| 207 | 0.01803 | 0.30 | 77.80 | 212.82 | 0.37 | [204823_at](https://www.affymetrix.com/LinkServlet?probeset=204823_at) | [NAV3](http://www.ncbi.nlm.nih.gov/entrez/query.fcgi?cmd=search&db=gene&term=NAV3) | neuron navigator 3 |
| 208 | 0.03333 | 0.34 | 422.78 | 1160.89 | 0.36 | [212354_at](https://www.affymetrix.com/LinkServlet?probeset=212354_at) | [SULF1](http://www.ncbi.nlm.nih.gov/entrez/query.fcgi?cmd=search&db=gene&term=SULF1) | sulfatase 1 |
| 209 | 0.01776 | 0.30 | 271.37 | 749.32 | 0.36 | [202006_at](https://www.affymetrix.com/LinkServlet?probeset=202006_at) | [PTPN12](http://www.ncbi.nlm.nih.gov/entrez/query.fcgi?cmd=search&db=gene&term=PTPN12) | protein tyrosine phosphatase, non-receptor type 12 |
| 210 | 0.00904 | 0.27 | 19.29 | 53.27 | 0.36 | [204311_at](https://www.affymetrix.com/LinkServlet?probeset=204311_at) | [ATP1B2](http://www.ncbi.nlm.nih.gov/entrez/query.fcgi?cmd=search&db=gene&term=ATP1B2) | ATPase, Na+/K+ transporting, beta 2 polypeptide |
| 211 | 0.01528 | 0.30 | 41.17 | 114.23 | 0.36 | [213222_at](https://www.affymetrix.com/LinkServlet?probeset=213222_at) | [PLCB1](http://www.ncbi.nlm.nih.gov/entrez/query.fcgi?cmd=search&db=gene&term=PLCB1) | phospholipase C, beta 1 (phosphoinositide-specific) |
| 212 | 0.02572 | 0.33 | 942.59 | 2615.33 | 0.36 | [226119_at](https://www.affymetrix.com/LinkServlet?probeset=226119_at) | [PCMTD1](http://www.ncbi.nlm.nih.gov/entrez/query.fcgi?cmd=search&db=gene&term=PCMTD1) | protein-L-isoaspartate (D-aspartate) O-methyltransferase domain containing 1 |
| 213 | 0.02530 | 0.33 | 19.85 | 55.35 | 0.36 | [226847_at](https://www.affymetrix.com/LinkServlet?probeset=226847_at) | [FST](http://www.ncbi.nlm.nih.gov/entrez/query.fcgi?cmd=search&db=gene&term=FST) | follistatin |
| 214 | 0.01931 | 0.31 | 90.06 | 251.75 | 0.36 | [207624_s_at](https://www.affymetrix.com/LinkServlet?probeset=207624_s_at) | [RPGR](http://www.ncbi.nlm.nih.gov/entrez/query.fcgi?cmd=search&db=gene&term=RPGR) | retinitis pigmentosa GTPase regulator |
| 215 | 0.00852 | 0.27 | 52.01 | 145.95 | 0.36 | [202660_at](https://www.affymetrix.com/LinkServlet?probeset=202660_at) | [ITPR2](http://www.ncbi.nlm.nih.gov/entrez/query.fcgi?cmd=search&db=gene&term=ITPR2) | inositol 1,4,5-triphosphate receptor, type 2 |
| 216 | 0.01228 | 0.29 | 301.03 | 845.01 | 0.36 | [212764_at](https://www.affymetrix.com/LinkServlet?probeset=212764_at) | [ZEB1](http://www.ncbi.nlm.nih.gov/entrez/query.fcgi?cmd=search&db=gene&term=ZEB1) | zinc finger E-box binding homeobox 1 |
| 217 | 0.02747 | 0.33 | 561.74 | 1583.03 | 0.35 | [225394_s_at](https://www.affymetrix.com/LinkServlet?probeset=225394_s_at) | [ZCRB1](http://www.ncbi.nlm.nih.gov/entrez/query.fcgi?cmd=search&db=gene&term=ZCRB1) | zinc finger CCHC-type and RNA binding motif 1 |
| 218 | 0.02745 | 0.33 | 214.70 | 607.31 | 0.35 | [202388_at](https://www.affymetrix.com/LinkServlet?probeset=202388_at) | [RGS2](http://www.ncbi.nlm.nih.gov/entrez/query.fcgi?cmd=search&db=gene&term=RGS2) | regulator of G-protein signaling 2, 24kDa |
| 219 | 0.01712 | 0.30 | 60.12 | 171.01 | 0.35 | [202196_s_at](https://www.affymetrix.com/LinkServlet?probeset=202196_s_at) | [DKK3](http://www.ncbi.nlm.nih.gov/entrez/query.fcgi?cmd=search&db=gene&term=DKK3) | dickkopf homolog 3 (Xenopus laevis) |
| 220 | 0.02410 | 0.32 | 193.98 | 554.19 | 0.35 | [203387_s_at](https://www.affymetrix.com/LinkServlet?probeset=203387_s_at) | [TBC1D4](http://www.ncbi.nlm.nih.gov/entrez/query.fcgi?cmd=search&db=gene&term=TBC1D4) | TBC1 domain family, member 4 |
| 221 | 0.01182 | 0.28 | 182.50 | 522.20 | 0.35 | [221127_s_at](https://www.affymetrix.com/LinkServlet?probeset=221127_s_at) | [RIG](http://www.ncbi.nlm.nih.gov/entrez/query.fcgi?cmd=search&db=gene&term=RIG) | regulated in glioma |
| 222 | 0.00779 | 0.27 | 21.95 | 62.95 | 0.35 | [226103_at](https://www.affymetrix.com/LinkServlet?probeset=226103_at) | [NEXN](http://www.ncbi.nlm.nih.gov/entrez/query.fcgi?cmd=search&db=gene&term=NEXN) | nexilin (F actin binding protein) |
| 223 | 0.03300 | 0.34 | 361.10 | 1038.39 | 0.35 | [211948_x_at](https://www.affymetrix.com/LinkServlet?probeset=211948_x_at) | [BAT2D1](http://www.ncbi.nlm.nih.gov/entrez/query.fcgi?cmd=search&db=gene&term=BAT2D1) | BAT2 domain containing 1 |
| 224 | 0.02107 | 0.32 | 273.62 | 788.96 | 0.35 | [212158_at](https://www.affymetrix.com/LinkServlet?probeset=212158_at) | [SDC2](http://www.ncbi.nlm.nih.gov/entrez/query.fcgi?cmd=search&db=gene&term=SDC2) | syndecan 2 |
| 225 | 0.00244 | 0.22 | 45.89 | 132.37 | 0.35 | [225142_at](https://www.affymetrix.com/LinkServlet?probeset=225142_at) | [JHDM1D](http://www.ncbi.nlm.nih.gov/entrez/query.fcgi?cmd=search&db=gene&term=JHDM1D) | jumonji C domain containing histone demethylase 1 homolog D (S. cerevisiae) |
| 226 | 0.03225 | 0.34 | 313.70 | 909.46 | 0.34 | [209732_at](https://www.affymetrix.com/LinkServlet?probeset=209732_at) | [CLEC2B](http://www.ncbi.nlm.nih.gov/entrez/query.fcgi?cmd=search&db=gene&term=CLEC2B) | C-type lectin domain family 2, member B |
| 227 | 0.00788 | 0.27 | 24.91 | 72.56 | 0.34 | [204401_at](https://www.affymetrix.com/LinkServlet?probeset=204401_at) | [KCNN4](http://www.ncbi.nlm.nih.gov/entrez/query.fcgi?cmd=search&db=gene&term=KCNN4) | potassium intermediate/small conductance calcium-activated channel, subfamily N, member 4 |
| 228 | 0.03317 | 0.34 | 107.68 | 316.70 | 0.34 | [226677_at](https://www.affymetrix.com/LinkServlet?probeset=226677_at) | [ZNF521](http://www.ncbi.nlm.nih.gov/entrez/query.fcgi?cmd=search&db=gene&term=ZNF521) | zinc finger protein 521 |
| 229 | 0.03100 | 0.34 | 216.09 | 636.53 | 0.34 | [230083_at](https://www.affymetrix.com/LinkServlet?probeset=230083_at) | [USP53](http://www.ncbi.nlm.nih.gov/entrez/query.fcgi?cmd=search&db=gene&term=USP53) | ubiquitin specific peptidase 53 |
| 230 | 0.03896 | 0.36 | 151.20 | 448.82 | 0.34 | [208994_s_at](https://www.affymetrix.com/LinkServlet?probeset=208994_s_at) | [PPIG](http://www.ncbi.nlm.nih.gov/entrez/query.fcgi?cmd=search&db=gene&term=PPIG) | peptidylprolyl isomerase G (cyclophilin G) |
| 231 | 0.04191 | 0.37 | 151.83 | 451.89 | 0.34 | [229584_at](https://www.affymetrix.com/LinkServlet?probeset=229584_at) | [LRRK2](http://www.ncbi.nlm.nih.gov/entrez/query.fcgi?cmd=search&db=gene&term=LRRK2) | leucine-rich repeat kinase 2 |
| 232 | 0.00556 | 0.25 | 25.62 | 76.29 | 0.34 | [221261_x_at](https://www.affymetrix.com/LinkServlet?probeset=221261_x_at) | [MAGED4B](http://www.ncbi.nlm.nih.gov/entrez/query.fcgi?cmd=search&db=gene&term=MAGED4B) | melanoma antigen family D, 4B |
| 233 | 0.01826 | 0.31 | 339.93 | 1015.64 | 0.33 | [201012_at](https://www.affymetrix.com/LinkServlet?probeset=201012_at) | [ANXA1](http://www.ncbi.nlm.nih.gov/entrez/query.fcgi?cmd=search&db=gene&term=ANXA1) | annexin A1 |
| 234 | 0.02587 | 0.33 | 484.40 | 1455.62 | 0.33 | [214055_x_at](https://www.affymetrix.com/LinkServlet?probeset=214055_x_at) | [BAT2D1](http://www.ncbi.nlm.nih.gov/entrez/query.fcgi?cmd=search&db=gene&term=BAT2D1) | BAT2 domain containing 1 |
| 235 | 0.03080 | 0.34 | 321.25 | 966.47 | 0.33 | [224840_at](https://www.affymetrix.com/LinkServlet?probeset=224840_at) | [FKBP5](http://www.ncbi.nlm.nih.gov/entrez/query.fcgi?cmd=search&db=gene&term=FKBP5) | FK506 binding protein 5 |
| 236 | 0.00687 | 0.26 | 493.58 | 1485.73 | 0.33 | [225171_at](https://www.affymetrix.com/LinkServlet?probeset=225171_at) | [ARHGAP18](http://www.ncbi.nlm.nih.gov/entrez/query.fcgi?cmd=search&db=gene&term=ARHGAP18) | Rho GTPase activating protein 18 |
| 237 | 0.02978 | 0.34 | 8.01 | 24.17 | 0.33 | [1557122_s_at](https://www.affymetrix.com/LinkServlet?probeset=1557122_s_at) | [GABRB2](http://www.ncbi.nlm.nih.gov/entrez/query.fcgi?cmd=search&db=gene&term=GABRB2) | gamma-aminobutyric acid (GABA) A receptor, beta 2 |
| 238 | 0.01188 | 0.29 | 289.11 | 872.07 | 0.33 | [202990_at](https://www.affymetrix.com/LinkServlet?probeset=202990_at) | [PYGL](http://www.ncbi.nlm.nih.gov/entrez/query.fcgi?cmd=search&db=gene&term=PYGL) | phosphorylase, glycogen, liver |
| 239 | 0.01908 | 0.31 | 98.54 | 299.23 | 0.33 | [222162_s_at](https://www.affymetrix.com/LinkServlet?probeset=222162_s_at) | [ADAMTS1](http://www.ncbi.nlm.nih.gov/entrez/query.fcgi?cmd=search&db=gene&term=ADAMTS1) | ADAM metallopeptidase with thrombospondin type 1 motif, 1 |
| 240 | 0.00935 | 0.27 | 173.96 | 534.58 | 0.33 | [224847_at](https://www.affymetrix.com/LinkServlet?probeset=224847_at) | [CDK6](http://www.ncbi.nlm.nih.gov/entrez/query.fcgi?cmd=search&db=gene&term=CDK6) | cyclin-dependent kinase 6 |
| 241 | 0.01986 | 0.31 | 101.64 | 312.72 | 0.33 | [228335_at](https://www.affymetrix.com/LinkServlet?probeset=228335_at) | [CLDN11](http://www.ncbi.nlm.nih.gov/entrez/query.fcgi?cmd=search&db=gene&term=CLDN11) | claudin 11 |
| 242 | 0.03565 | 0.35 | 20.20 | 63.11 | 0.32 | [218451_at](https://www.affymetrix.com/LinkServlet?probeset=218451_at) | [CDCP1](http://www.ncbi.nlm.nih.gov/entrez/query.fcgi?cmd=search&db=gene&term=CDCP1) | CUB domain containing protein 1 |
| 243 | 0.00868 | 0.27 | 18.04 | 56.81 | 0.32 | [229649_at](https://www.affymetrix.com/LinkServlet?probeset=229649_at) | [NRXN3](http://www.ncbi.nlm.nih.gov/entrez/query.fcgi?cmd=search&db=gene&term=NRXN3) | neurexin 3 |
| 244 | 0.01663 | 0.30 | 51.36 | 161.79 | 0.32 | [223796_at](https://www.affymetrix.com/LinkServlet?probeset=223796_at) | [CNTNAP3](http://www.ncbi.nlm.nih.gov/entrez/query.fcgi?cmd=search&db=gene&term=CNTNAP3) | contactin associated protein-like 3 |
| 245 | 0.02735 | 0.33 | 368.77 | 1163.83 | 0.32 | [201505_at](https://www.affymetrix.com/LinkServlet?probeset=201505_at) | [LAMB1](http://www.ncbi.nlm.nih.gov/entrez/query.fcgi?cmd=search&db=gene&term=LAMB1) | laminin, beta 1 |
| 246 | 0.00405 | 0.24 | 49.74 | 157.76 | 0.32 | [201641_at](https://www.affymetrix.com/LinkServlet?probeset=201641_at) | [BST2](http://www.ncbi.nlm.nih.gov/entrez/query.fcgi?cmd=search&db=gene&term=BST2) | bone marrow stromal cell antigen 2 |
| 247 | 0.01572 | 0.30 | 300.88 | 961.01 | 0.31 | [212177_at](https://www.affymetrix.com/LinkServlet?probeset=212177_at) | [SFRS18](http://www.ncbi.nlm.nih.gov/entrez/query.fcgi?cmd=search&db=gene&term=SFRS18) | splicing factor, arginine/serine-rich 18 |
| 248 | 0.01457 | 0.29 | 43.09 | 137.70 | 0.31 | [204422_s_at](https://www.affymetrix.com/LinkServlet?probeset=204422_s_at) | [FGF2](http://www.ncbi.nlm.nih.gov/entrez/query.fcgi?cmd=search&db=gene&term=FGF2) | fibroblast growth factor 2 (basic) |
| 249 | 0.00570 | 0.25 | 31.14 | 99.94 | 0.31 | [228749_at](https://www.affymetrix.com/LinkServlet?probeset=228749_at) | [ZDBF2](http://www.ncbi.nlm.nih.gov/entrez/query.fcgi?cmd=search&db=gene&term=ZDBF2) | zinc finger, DBF-type containing 2 |
| 250 | 0.04333 | 0.37 | 166.61 | 538.44 | 0.31 | [212027_at](https://www.affymetrix.com/LinkServlet?probeset=212027_at) | [RBM25](http://www.ncbi.nlm.nih.gov/entrez/query.fcgi?cmd=search&db=gene&term=RBM25) | RNA binding motif protein 25 |
| 251 | 0.00194 | 0.21 | 45.16 | 146.04 | 0.31 | [204030_s_at](https://www.affymetrix.com/LinkServlet?probeset=204030_s_at) | [SCHIP1](http://www.ncbi.nlm.nih.gov/entrez/query.fcgi?cmd=search&db=gene&term=SCHIP1) | schwannomin interacting protein 1 |
| 252 | 0.01248 | 0.29 | 137.60 | 450.56 | 0.31 | [226621_at](https://www.affymetrix.com/LinkServlet?probeset=226621_at) | [FGG](http://www.ncbi.nlm.nih.gov/entrez/query.fcgi?cmd=search&db=gene&term=FGG) | fibrinogen gamma chain |
| 253 | 0.02863 | 0.33 | 30.39 | 104.83 | 0.29 | [201596_x_at](https://www.affymetrix.com/LinkServlet?probeset=201596_x_at) | [KRT18](http://www.ncbi.nlm.nih.gov/entrez/query.fcgi?cmd=search&db=gene&term=KRT18) | keratin 18 |
| 254 | 0.00446 | 0.24 | 158.57 | 547.69 | 0.29 | [202748_at](https://www.affymetrix.com/LinkServlet?probeset=202748_at) | [GBP2](http://www.ncbi.nlm.nih.gov/entrez/query.fcgi?cmd=search&db=gene&term=GBP2) | guanylate binding protein 2, interferon-inducible |
| 255 | 0.02129 | 0.32 | 13.81 | 48.14 | 0.29 | [202036_s_at](https://www.affymetrix.com/LinkServlet?probeset=202036_s_at) | [SFRP1](http://www.ncbi.nlm.nih.gov/entrez/query.fcgi?cmd=search&db=gene&term=SFRP1) | secreted frizzled-related protein 1 |
| 256 | 0.01676 | 0.30 | 639.25 | 2265.64 | 0.28 | [208835_s_at](https://www.affymetrix.com/LinkServlet?probeset=208835_s_at) | [CROP](http://www.ncbi.nlm.nih.gov/entrez/query.fcgi?cmd=search&db=gene&term=CROP) | cisplatin resistance-associated overexpressed protein |
| 257 | 0.00444 | 0.24 | 25.54 | 90.57 | 0.28 | [1555564_a_at](https://www.affymetrix.com/LinkServlet?probeset=1555564_a_at) | [CFI](http://www.ncbi.nlm.nih.gov/entrez/query.fcgi?cmd=search&db=gene&term=CFI) | complement factor I |
| 258 | 0.03741 | 0.36 | 53.08 | 189.15 | 0.28 | [204595_s_at](https://www.affymetrix.com/LinkServlet?probeset=204595_s_at) | [STC1](http://www.ncbi.nlm.nih.gov/entrez/query.fcgi?cmd=search&db=gene&term=STC1) | stanniocalcin 1 |
| 259 | 0.01979 | 0.31 | 13.92 | 50.04 | 0.28 | [204471_at](https://www.affymetrix.com/LinkServlet?probeset=204471_at) | [GAP43](http://www.ncbi.nlm.nih.gov/entrez/query.fcgi?cmd=search&db=gene&term=GAP43) | growth associated protein 43 |
| 260 | 0.01078 | 0.28 | 298.04 | 1076.28 | 0.28 | [200795_at](https://www.affymetrix.com/LinkServlet?probeset=200795_at) | [SPARCL1](http://www.ncbi.nlm.nih.gov/entrez/query.fcgi?cmd=search&db=gene&term=SPARCL1) | SPARC-like 1 (hevin) |
| 261 | 0.04331 | 0.37 | 37.66 | 136.14 | 0.28 | [217901_at](https://www.affymetrix.com/LinkServlet?probeset=217901_at) | [DSG2](http://www.ncbi.nlm.nih.gov/entrez/query.fcgi?cmd=search&db=gene&term=DSG2) | desmoglein 2 |
| 262 | 0.01245 | 0.29 | 75.73 | 276.31 | 0.27 | [228933_at](https://www.affymetrix.com/LinkServlet?probeset=228933_at) | [NHS](http://www.ncbi.nlm.nih.gov/entrez/query.fcgi?cmd=search&db=gene&term=NHS) | Nance-Horan syndrome (congenital cataracts and dental anomalies) |
| 263 | 0.01931 | 0.31 | 34.83 | 127.39 | 0.27 | [207012_at](https://www.affymetrix.com/LinkServlet?probeset=207012_at) | [MMP16](http://www.ncbi.nlm.nih.gov/entrez/query.fcgi?cmd=search&db=gene&term=MMP16) | matrix metallopeptidase 16 (membrane-inserted) |
| 264 | 0.00320 | 0.23 | 398.71 | 1481.86 | 0.27 | [230746_s_at](https://www.affymetrix.com/LinkServlet?probeset=230746_s_at) | [STC1](http://www.ncbi.nlm.nih.gov/entrez/query.fcgi?cmd=search&db=gene&term=STC1) | stanniocalcin 1 |
| 265 | 0.00444 | 0.24 | 163.24 | 607.40 | 0.27 | [204597_x_at](https://www.affymetrix.com/LinkServlet?probeset=204597_x_at) | [STC1](http://www.ncbi.nlm.nih.gov/entrez/query.fcgi?cmd=search&db=gene&term=STC1) | stanniocalcin 1 |
| 266 | 0.02296 | 0.32 | 30.40 | 114.41 | 0.27 | [206766_at](https://www.affymetrix.com/LinkServlet?probeset=206766_at) | [ITGA10](http://www.ncbi.nlm.nih.gov/entrez/query.fcgi?cmd=search&db=gene&term=ITGA10) | integrin, alpha 10 |
| 267 | 0.00187 | 0.21 | 60.75 | 244.42 | 0.25 | [228728_at](https://www.affymetrix.com/LinkServlet?probeset=228728_at) | [C7orf58](http://www.ncbi.nlm.nih.gov/entrez/query.fcgi?cmd=search&db=gene&term=C7orf58) | chromosome 7 open reading frame 58 |
| 268 | 0.02727 | 0.33 | 26.68 | 108.63 | 0.25 | [203895_at](https://www.affymetrix.com/LinkServlet?probeset=203895_at) | [PLCB4](http://www.ncbi.nlm.nih.gov/entrez/query.fcgi?cmd=search&db=gene&term=PLCB4) | phospholipase C, beta 4 |
| 269 | 0.00042 | 0.19 | 283.15 | 1159.00 | 0.24 | [224657_at](https://www.affymetrix.com/LinkServlet?probeset=224657_at) | [ERRFI1](http://www.ncbi.nlm.nih.gov/entrez/query.fcgi?cmd=search&db=gene&term=ERRFI1) | ERBB receptor feedback inhibitor 1 |
| 270 | 0.01959 | 0.31 | 30.09 | 125.37 | 0.24 | [206805_at](https://www.affymetrix.com/LinkServlet?probeset=206805_at) | [SEMA3A](http://www.ncbi.nlm.nih.gov/entrez/query.fcgi?cmd=search&db=gene&term=SEMA3A) | sema domain, immunoglobulin domain (Ig), short basic domain, secreted, (semaphorin) 3A |
| 271 | 0.04561 | 0.37 | 39.75 | 166.31 | 0.24 | [204475_at](https://www.affymetrix.com/LinkServlet?probeset=204475_at) | [MMP1](http://www.ncbi.nlm.nih.gov/entrez/query.fcgi?cmd=search&db=gene&term=MMP1) | matrix metallopeptidase 1 (interstitial collagenase) |
| 272 | 0.00125 | 0.20 | 18.33 | 81.66 | 0.22 | [205609_at](https://www.affymetrix.com/LinkServlet?probeset=205609_at) | [ANGPT1](http://www.ncbi.nlm.nih.gov/entrez/query.fcgi?cmd=search&db=gene&term=ANGPT1) | angiopoietin 1 |
| 273 | 0.00105 | 0.20 | 54.17 | 247.83 | 0.22 | [203854_at](https://www.affymetrix.com/LinkServlet?probeset=203854_at) | [CFI](http://www.ncbi.nlm.nih.gov/entrez/query.fcgi?cmd=search&db=gene&term=CFI) | complement factor I |
| 274 | 0.00067 | 0.20 | 45.60 | 222.70 | 0.20 | [203786_s_at](https://www.affymetrix.com/LinkServlet?probeset=203786_s_at) | [TPD52L1](http://www.ncbi.nlm.nih.gov/entrez/query.fcgi?cmd=search&db=gene&term=TPD52L1) | tumor protein D52-like 1 |
| 275 | 0.02051 | 0.31 | 73.36 | 375.97 | 0.20 | [227145_at](https://www.affymetrix.com/LinkServlet?probeset=227145_at) | [LOXL4](http://www.ncbi.nlm.nih.gov/entrez/query.fcgi?cmd=search&db=gene&term=LOXL4) | lysyl oxidase-like 4 |
| 276 | 0.04338 | 0.37 | 49.45 | 264.73 | 0.19 | [202376_at](https://www.affymetrix.com/LinkServlet?probeset=202376_at) | [SERPINA3](http://www.ncbi.nlm.nih.gov/entrez/query.fcgi?cmd=search&db=gene&term=SERPINA3) | serpin peptidase inhibitor, clade A (alpha-1 antiproteinase, antitrypsin), member 3 |
| 277 | 0.00465 | 0.24 | 108.97 | 617.51 | 0.18 | [223614_at](https://www.affymetrix.com/LinkServlet?probeset=223614_at) | [C8orf57](http://www.ncbi.nlm.nih.gov/entrez/query.fcgi?cmd=search&db=gene&term=C8orf57) | chromosome 8 open reading frame 57 |
| 278 | 0.00993 | 0.28 | 48.07 | 376.46 | 0.13 | [202037_s_at](https://www.affymetrix.com/LinkServlet?probeset=202037_s_at) | [SFRP1](http://www.ncbi.nlm.nih.gov/entrez/query.fcgi?cmd=search&db=gene&term=SFRP1) | secreted frizzled-related protein 1 |
